# Quantifying future climate extreme indices: implications for sustainable urban development in West Africa, with a focus on the greater Accra region

**DOI:** 10.1007/s43621-024-00352-w

**Published:** 2024-07-29

**Authors:** Ebenezer Kwadwo Siabi, Edward Abingya Awafo, Amos Tiereyangn Kabobah, Nana Sarfo Agyeman Derkyi, Komlavi Akpoti, Geophrey Kwame Anornu, Mashael Yazdanie

**Affiliations:** 1 Earth Observation Research and Innovation Center (EORIC), University of Energy and Natural Resources, P.O. Box 214, Sunyani, Ghana; 2 Department of Agricultural and Bioresources Engineering, University of Energy and Natural Resources, P.O. Box 214, Sunyani, Ghana; 3 Department of Civil and Environmental Engineering, University of Energy and Natural Resources, P.O. Box 214, Sunyani, Ghana; 4 Department of Renewable Energy Engineering, University of Energy and Natural Resources, P.O. Box 214, Sunyani, Ghana; 5 International Relations Office, University of Energy and Natural Resources, P.O. Box 214, Sunyani, Ghana; 6 Regional Center for Energy and Environmental Sustainability, University of Energy and Natural Resources, P.O. Box 214, Sunyani, Ghana; 7 International Water Management Institute (IWMI), Accra, Ghana; 8 Department of Civil Engineering, Kwame Nkrumah University of Science and Technology, Kumasi, Ghana; 9 Urban Energy Systems Laboratory, Empa, Swiss Federal Laboratories for Materials Science and Technology, Überlandstrasse 129, 8600 Dübendorf, Switzerland

**Keywords:** Droughts, Floods, Heatwaves, Climate risk, SSP scenarios, Mega cities

## Abstract

Climate change leading to Climate extremes in the twenty-first century is more evident in megacities across the world, especially in West Africa. The Greater Accra region is one of the most populated regions in West Africa. As a result, the region has become more susceptible to climate extremes such as floods, heatwaves, and droughts. The study employed the Coupled Model Intercomparison Project 6 models in simulating climate extreme indices under the Shared Socioeconomic Pathway scenarios (SSPs) over West Africa between 1979 and 2059 as exemplified by the Greater Accra region. The study observed a generally weak drought in the historical period and expected to intensify especially under SSP585 in Greater Accra. For instance, continuous dry days (CDD) reveal an increasing trend under the SSPs. Similarly, the overall projected trend of CDD over West Africa reveals an increase signifying a more frequent and longer drought in the future. The flood indices revealed a surge in the intensity and duration of extreme precipitation events under the SSPs in the region. For instance, R99pTOT and Rx5days are expected to significantly increase under the SSPs with intensification under the SSP245, SSP370, and SSP585. A similar trend has been projected across West Africa, especially along the Guinean coast. The study foresees a gradual and intensifying rise in heatwave indices over the Greater Accra region. The warming and cooling indices reveal an increasing and decreasing trend respectively in the historical period as well as under the SSPs particularly within urban centers like Accra and Tema. Most West African countries are projected to observe more frequent warm days and nights with cold nights and days becoming less frequent. Expected effects of future climate extreme indices pose potential threats to the water, food, and energy systems as well as trigger recurrent floods and droughts over Greater Accra. The findings of the study are expected to inform climate policies and the nationally determined contribution of the Paris Agreement as well as address the sustainable development goal 11 (Sustainable cities) and 13 (Climate action) in West Africa.

## Introduction

The mean temperature of large regions in Africa is expected to surpass the global threshold of 2 °C by 2050 and rise up to 2.6–4.8 under the medium- and high-emissions [1]. Despite Africa’s contribution to total global greenhouse gas (GHG) emissions being relatively small, the effects on agriculture, water resources, and ecosystems are expected to be detrimental due to the low adaptive capacity [2]. Since the fourth and fifth assessment reports (AR4 and AR5 respectively), the effects of climate change (CC) have escalated, affecting the intensity, frequency, and duration of extreme events, some of which are projected to persist [3]. Extreme climate events (ECEs) (popularly termed extreme events) are amongst the key manifestations of CC in a region. Extreme event occurs when “one set counts the number of days when maximum daily temperature is above a relative threshold defined as the 90th or higher percentile of maximum daily temperature for the calendar day over a base period” [3]. Based on this definition, an event can occur at any time of the year with varying impacts depending on the season. The other set records the number of days the maximum diurnal temperature exceeds an absolute threshold of 35 °C, since going above this temperature occasionally has negative health effects. However, these effects can differ depending on the environment and whether or not the population and ecosystems are accustomed to such temperatures [3].

Over the last several decades, changes in extreme precipitation and temperature events, together with dry conditions, have resulted in a series of ECEs [4]. Studies on extreme temperatures in Western and Southern Africa showed persistent warming between 1961 and 2000 [5]. For instance, the study of Mouhamed et al. [4] identified a generic warming trend over significant areas in West Africa between 1960 and 2010. The study further revealed more frequent extreme precipitation events in the West African Sahel in the last decade compared to 1961–1990. Gbode et al. [6] also showed a surge in the amount of cool nights and warm days between 1960 and 2007 in Kano, Nigeria. Marginal increases were evident in the annual total precipitation whereas the number of extremely wet days increased significantly. Moreover, drought (which is an intrinsic attribute of the subregion) is among the main causes of poverty, mortality, migration, etc. in West Africa [7]. For instance, the 1970s and 1980s extreme drought in West Africa resulted in water shortages for agricultural and socio-economic use [8, 9]. As a result, famine predominated the subregion leading to the death of about 250,000 people as well as over 3 million animals worth 400 million dollars [10]. In Ghana, a series of bushfires occurred as a result of the extreme drought and resulted in the loss of lives, agricultural lands as well as property. ECEs in Ghana especially in Greater Accra have surged significantly in recent decades. Some salient examples such as the extreme flood events in 2001, 2007, 2011, and 2015 [11, 12]. According to EM-DAT report as cited in [9] over 3,885,695 people have been affected and $108,200,00 worth of damages caused as a result of flooding in Greater Accra. The majority of existing studies attributed the cause of flood and heatwaves in the Greater Accra region to anthropogenic causes such as rural–urban migration resulting in urban sprawl [11, 13–16]. Other studies hammered on the weak institutional structures leading to indiscriminate building of structures and disposal of waste in waterways [17, 18]. Despite this, ECEs may also be a cause. As such, the projections of climate extreme indices provide an essential understanding and knowledge for planning the risks associated with these hazards, especially in water, food, and energy resources management and civil defense.

There are limited studies on the impacts of climate extreme indices on megacities in West Africa such as the Greater Accra region. Thus, detailed climate extreme indices study at a mega city level (such as Greater Accra), especially under the Shared Socioeconomic Pathways scenarios (SSPs), is still lacking. For instance, various studies have explored the effects of climate change in the Greater Accra region [16–19], however, these studies are limited to historical climate extremes events. The future projection of climate extreme indices over the Greater Accra region was not assessed. The quantification of future climate extreme indices is critical to adequately prepare and formulate adaptation and mitigation strategies. As such, Global Climate Models (GCMs) and Regional Climate models (RCMs) are the commonly used tools for future climate projections. Aside from this, they are also used to explore the variations of historical and future climate conditions [20, 21]. Policymakers, however, are often drawn to simulation with a fine resolution of 1–50 km or even more [22]. To close this gap, dynamic and statistical downscaling methods are used. The new Coupled Intercomparison Project extreme Phase 6 (CMIP 6) models reproduce similar outputs as those predicted by the CMIP5 models with high confidence [3]. However, the performances of CMIP6 and CMIP5 have been evaluated by different studies revealing the enhancement in the CMIP6 GCMs relative to the CMIP5 [23–25].

This study gains significance when contextualized with previous research conducted in the region. Presently, there is a gap in the examination of climate extreme indices under the SSPs in the Greater Accra region. This void is noteworthy, as climate extremes are significantly influenced by the ongoing socio-economic activities in a given area. Anticipated increases in carbon dioxide (CO_2_), methane (CH_4_), and other emissions, driven by future population growth, further emphasize the potential exacerbation of climate extremes in the region. Against this background, the study aims to simulate climate extreme indices in the Greater Accra region from 1979 to 2059 under different SSPs. The study further analyses the historical and future spatio-temporal distribution/characteristics of climate extreme indices and their impacts over the Greater Accra region under different SSPs. The findings of the study are expected to enhance the quality of decision-making services in response to climate change. It also provides scientific references for the socioeconomic, ecological environment, and security as well as development planning in the region.

## Materials and methods

### Description of the study area

**Fig. 1:**
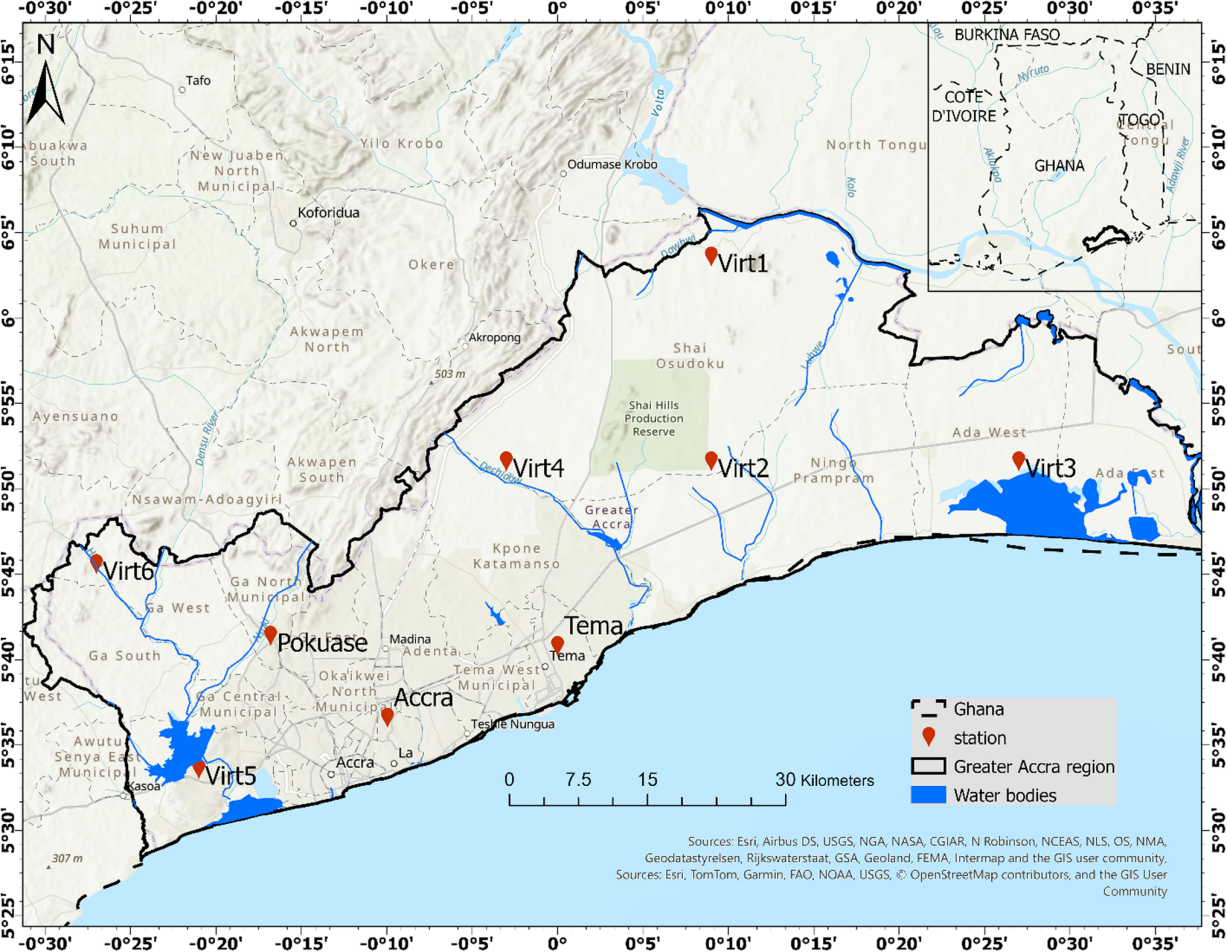
Study area map showing the various locations of stations used for the study

Figure 1 presents the map of the studied area. The Greater Accra Region is one of the sixteen administrative regions of Ghana, located on the southern coast of the country. It is situated between Latitude 5°30′ and 5°53′ North and Longitude 0°03′ and 0°25′ West. Greater Accra region is the smallest region in terms of area, covering an area of 3245 square kilometers, but is the most densely populated. The region's capital is Accra, which serves as the political and economic hub of the country. The region has a population of about 5,455,692 people [26]. Greater Accra is located in the anomalous dry equatorial climatic zone with a bimodal rainfall pattern and a prolonged dry season. The region falls within the southern belt of Ghana which is characterized by low temperatures [27]. The hottest months in the study area are February and March with an average monthly temperature of about 27 °C [28]. June to August are the coldest months with an average temperature of 21 °C. The dual peak rainfall seasons occur from March to July and September to November, bringing an annual rainfall of approximately 780–1200 mm [29]. The average rainfall is approximately 812 mm as recorded by the Ghana Meteorological Agency [29]. In terms of economic activity, the Greater Accra Region is a major contributor to Ghana's economy, accounting for about one-third of the country's GDP. The region is home to a variety of industries, including manufacturing, trade, and services, and is also a major center for banking and finance [30–32]. Overall, the Greater Accra Region is a vibrant and dynamic part of Ghana, with a diverse population and thriving economy. To address data scarcity challenges in certain areas of the Greater Accra region, six virtual stations were employed to serve as representations for these specific locations (Fig. 1).

### Station data and CMIP6 models used

The study used Precipitation (Prcp) data, and Minimum (Tmin) and Maximum (Tmax) temperature attained from the Ghana meteorological agency. For the whole of Greater Accra, data for three stations (Accra, Pokuase, and Tema) were available. Data for the remaining areas within the Greater Accra region was unavailable. For this purpose, the study utilized the Princeton University Global Meteorological Forcing (PUGMF) dataset [33]. This data supplemented the remaining areas in Greater Accra without station data. Therefore, these areas were represented by virtual stations. The PUGMF data was utilized because it combines data from various sources such as global and station-based datasets. Inherent in the PUGMF datasets are contributing datasets, including the NCAR (National Center for Atmospheric Research Reanalysis), SRB (NASA Langley Surface Radiation Budget), CRU TS (Climate Research Unit Timeseries) 2.0, and NCEP (National Center for Environmental Prediction) datasets, and the GPCP (Global Precipitation Climatology Project), all integrated as ensembles. This increases the accuracy consistency and reliability, as well as reduces the uncertainty, of the PUGMF datasets. Moreover, the PUGMF dataset was used because previous studies have revealed its strong correlation with ground station data [33, 34].

Concerning the CMIP6 data used, six Global Climate Models (GCMs) (see Table 1) were utilized for the study [34]. The study employed the CMIP6 data as a result of its higher accuracy and resolution than the CMIP5. Again, the incorporation of the socio-economic variables, which has a strong connotation on CC, makes it a better choice for the study. Hence, the study examined four SSPs including SSP126, SSP245, SSP370, and SSP585) [46], spanning from 2015 to 2059, in comparison to the observed periods of 1970 to 2014 and 1970 to 2010 for observed and virtual stations, respectively. To capture uncertainties in future climate extreme indices, the multi-model ensemble was used to project climate extreme indices for precipitation and temperature in the Greater Accra region.

**Table 1:** Reference of selected CMIP6 dataset for the study [45]

Model	Institute	Country	Horizontal resolution
BCC-CSM2-MR	Beijing Climate Center Climate System Model	China	1.125° × 1.125°
IPSL-CM6A-LR	Institut Pierre-Simon Laplace (IPSL)	France	2.5° × 1.3°
MPI-ESM1-2-HR	Max Planck Institute for Meteorology	Germany	0.9° × 0.9°
MPI-ESM1-2-LR	Max Planck Institute for Meteorology	Germany	1.9° × 1.9°
CanESM5	Canadian Earth System Model	Canada	2.8° × 2.8°
MRI-ESM2-0	Meteorological Research Institute (MRI)	Japan	1.1° × 1.1°

### Climate model data for hydrologic modeling (CMhyd)

Statistical bias correction was done using the CMhyd. The model is specifically designed to correct biases in data obtained from GCMs and RCMs. Bias correction becomes necessary as a result of the inherently coarse resolution of GCM and RCM datasets. The bias correction procedure involves employing a transformation algorithm to predict the model's output. The assumption is that the algorithm and its parameterization for bias correction remain valid not only for current climate conditions but also for future conditions. CMhyd has found widespread application in various contexts [35–38]. The study used CMhyd because of its compatibility with CMIP6 data, enabling the simulation of historical and future climates. The CMhyd tool offers a choice of eight distinct bias-correction algorithms. However, a study by Zhang et al. [39] identified the distribution mapping algorithm as the most effective algorithm among five tested bias-correction algorithms. Therefore, the study used quantile mapping, also known as distribution mapping. This algorithm was selected because of its performance in aligning the distribution function of simulated GCM output with that of the station data, as demonstrated in various studies [40–43].

### Evaluation of model performance

Various evaluation metrics were employed in the study to evaluate the model performance. The accuracy of the bias-corrected model was assessed using RMSE (Root Mean Square Error) (Eq. 1), R^2^ (coefficient of determination) (Eq. 2), Pbias (percent bias) (Eq. 3), and NSE (Nash–Sutcliffe coefficient) (Eq. 4). RMSE functioned as an indicator of goodness-of-fit, representing the standard deviation between the observed and modeled data. A smaller RMSE signifies enhanced model performance. Additionally, the goodness-of-fit of the observed and modeled data was assessed using the R^2^, with closer proximity to one signifying enhanced model performance.

The NSE (− ∞ to 1) deems the model satisfactory when less than or equal to 0.5 and a very good fit when less than or equal to 0.7. The Pbias was used to assess whether the model underestimates or overestimates observed data. The model performance improves as Pbias moves closer to zero.

1 $$RMSE=\sqrt{\frac{{\sum }_{i=1}^{N}({Q}_{obs}-{Q}_{sim}{)}^{2}}{N}}$$

2 $${R}^{2}=1-\frac{{\sum }_{i=1}^{N}({Q}_{obs}-{Q}_{sim}{)}^{2}}{{\sum }_{i=1}^{N}({Q}_{obs}-{Q}_{obs}{)}^{2}}$$

3 $$PBIAS=\frac{{\sum }_{i=1}^{N}({Q}_{obs}-{Q}_{sim})}{{\sum }_{i=1}^{N}({Q}_{obs})}\times 100$$

4 $$NSE=1-\left[\frac{{\sum }_{i=1}^{N}({Q}_{obs}-{Q}_{sim}{)}^{2}}{{\sum }_{i=1}^{N}({Q}_{obs}-{\overline{Q} }_{obs}{)}^{2}}\right]$$

where ${Q}_{obs}$ is the observed data, ${Q}_{sim}$ is the predicted data, ${\bar{Q}}_{obs}$ is the mean observed data, $N$ is the number of observations.

### Climate indices

The study employed the multi-model bias-corrected ensemble from the CMhyd. The data spans from 1970 to 2014 for the historical period and 2015–2059 for the future period. The study selected a total of 15 indices specified by the Expert Team on Sector-specific Climate Indices (ET-SCI). The selected indices were categorized (into drought, flood, and heatwave) according to their contribution to the frequently occurring climate extreme events that impact various essential sectors in the Greater Accra region (see Table 2). The ClimPACT2 software developed by the ET-SCI [44] was used for the computation of the selected indices. It should be noted that the ET-SCI/ETCCDI indices only characterize moderate extreme events [45]. The current study focuses on moderate extreme events rather than rare extreme events of the Generalized Extreme Value Theory (GEVT). Moreover, due to moderate extreme events occurring at least once per year, their results are more robust compared to the rare extreme events [46].

**Table 2:** Selected extreme indices for the study

Index	Description	Definition	Units
*Drought*			
CDD	Consecutive Dry Days	Maximum number of consecutive dry days (when Prcp < 1.0 mm)	days
SPI	Standardized Precipitation Index	Measure of drought” using the Standardized Precipitation Index on time scales of 3, 6, and 12 months	unitless
SPEI	Standardized Precipitation Evapotranspiration Index	Measure of drought using the Standardized Precipitation Evapotranspiration Index on time scales of 3, 6 and 12 months	unitless
*Flooding*			
CWD	Consecutive Wet Days	Maximum annual number of consecutive wet days (when Prcp ≥ 1.0 mm)	days
PRCPTOT	Annual total wet-day Prcp	Sum of daily Prcp ≥ 1.0 mm	mm
R20mm	Number of very heavy rain days	Number of days when Prcp ≥ 20 mm	days
R99pTOT	Contribution from extremely wet days	100*r99p/PRCPTOT	%
R99p	Total annual Prcp from very heavy rain days	Annual sum of daily Prcp > 99th percentile	mm
Rx5day	Maximum 5-day Prcp total	Maximum amount of rain that falls in five consecutive days	mm
*Heatwave*			
Tx10p	Amount of cool days	Percentage of days when Tmax < 10th percentile	%
Tx90p	Amount of hot days	Percentage of days when Tmax > 90th percentile	%
Tn10p	Amount of cold nights	Percentage of days when Tmin < 10th percentile	%
Tn90p	Amount of warm nights	Percentage of days when Tmin > 90th percentile	%
TXx	Max Tmax	Warmest daily Tmax	°C
TNx	Min Tmin	Coldest daily Tmin	°C
WSDI	Warm spell duration indicator	Annual number of days contributing to events where 6 or more consecutive days experience Tmax > 90th percentile	Days
CDDcold	Cooling Degree Days	A measure of the energy demand needed to cool a building	degree-days

The three daily datasets (Tmin, Tmax, and Prcp) were preprocessed according to the specifications of the ET-SCI and served as input for the ClimPACT2 package. Existing studies [47, 48] noted that quantifying the intensity, duration, and frequency of climate extremes can be achieved based on daily time series of precipitation and temperature.

Data quality control was undertaken to avoid unrealistic data such as days with negative or > 500 mm precipitation amount, days with Tmin ≥ Tmax, as well as Tmin and Tmax > six (6) standard deviations from the long-term (i.e., full dataset) mean value [49]. As such, the unrealistic/erroneous data were replaced with − 99.9 which is detected by ClimPACT2 package as missing data to avoid errors in the computation. Moreover, a homogeneity test was carried out on each time series under the data quality control. After implementing these control measures the ground station data was more than 85% consistent to proceed with the study. The computation process was executed for the baseline period, as well as the future periods. For future periods, the computation was performed based on scenarios (i.e., for SSP126, SSP245, SSP370, and SSP585). Table 3 shows the SPEI and SPI classification of moisture levels and their description.

**Table 3:** Indices contributing to drought [63, 64]

SPEI value	Moisture level
+ 2.0 and greater	Extremely wet
+ 1.5 to 1.99	Very wet
+ 1 to 1.49	Moderately wet
− 0.99 to 0.99	Near normal
− 1.49 to − 1.0	Moderately dry
− 1.99 to − 1.5	Severely dry
Less to − 2.0	Extremely dry

SPI	Classification
2.00 or more	Extremely wet
1.50 to 1.99	Severely wet
1.00 to 1.49	Moderately wet
0 to 0.99	Mildly wet
0 to − 0.99	Mild drought
− 1.00 to − 1.49	Moderate drought
− 1.50 to − 1.99	Severe drought
− 2 or less	Extreme drought

In estimating the daily degree-days, the ASHRAE estimation method was employed [50]. Daily cooling degree-days (CDDcold) are computed using Eq. 5:

5 $${{CDDcold}_{d}=\left(\frac{Tmax+Tmin}{2}-Tbase\right)}^{+}$$

where Tmax is the daily maximum temperature, Tmin is the daily minimum temperature, The ‘+’ superscript implies that only positive values of the bracketed quantity are considered in the sum.

Tbase is the base temperature (this is the basic notation that explains the association between climate, building construction, occupancy, and the energy flow path in a building). Therefore, the study employed 25 °C as the average room temperature in Greater Accra where the building needs no cooling or heating. For standardization, the Celsius-based cooling degree day with a base temperature of 25 °C was converted into the corresponding degree days. Given as a proper SI unit, a quantity of Kelvin second (K s) is four orders of magnitude higher than the corresponding degree day. Therefore, 1 °C-day is equal to 8.64 × 10^4^ K s.

### Trend analysis

The study used the Modified Mann–Kendall test [51] in quantifying and identifying the trends in the selected indices. Unlike the Mann–Kendall test, the modified Mann–Kendall test (MMKT) addresses the auto-correlation issues in a time series. The MMKT demonstrates a robust tool for detecting monotonic trends [52] by considering a test statistic Z with Z > 0 showing increasing trends and vice versa. Validation is done by the direction of the slope with negative values representing decreasing trends and vice versa. The Sen's slope coefficient (SSC) shows whether there is a negative, positive, or no trend in the variable. Moreover, a positive Sen's slope coefficient shows a trend line sloping upward over time and vice versa for a negative SSC. It is worth noting that the degree of the coefficient indicates the strength of the trend. Larger coefficients indicate a stronger trend whereas smaller coefficients indicate a weaker trend. The corrected Zc and new *P*-value indicate the magnitude and significance of the trend. The Corrected Zc values greater than 1.96 or less than − 1.96 indicate a statistically significant trend at the 95% confidence level. The 95% confidence interval with *P*-values less than 0.05 and z value of ± 1.96 was used to examine the significance of trends. See [51, 53] for further details concerning the computation of MMKT and the TheilSen estimator, which has been applied extensively in hydrology and meteorology [54, 55].

Hamed and Rao [56] modified the VAR(S) statistics as:

6 $$VAR\left(S\right)=\left(\frac{n\left(n-1\right)\left(2n+5\right)}{18}\right)\times \left(\frac{n}{{n}_{e}^{*}}\right)$$

where the correction factor $\left(\frac{n}{{n}_{e}^{*}}\right)$ is adjusted to the autocorrelated data as:

7 $$\left(\frac{n}{{n}_{e}^{*}}\right)=1+\left(\frac{2}{{n}^{3}-{3n}^{2}+2n}\right){\sum }_{f=1}^{n-1}\left(n-f\right)\left(n-f-1\right)\left(n-f-2\right){\rho }_{e}\left(f\right)$$

where ${\rho }_{e}\left(f\right)$ denotes the autocorrelation between ranks of observation and can be computed as;

8 $${\rho }_{e}\left(f\right)=2sin\left(\frac{\pi }{6}\right){\rho }_{e}\left(f\right)$$

The Sen’s Slope Estimator (Y) is the median of N values of ${Y}_{i}$ [54]

9 $${\text{Where}}\quad Y_i = \frac{x_k - x_j }{{k - j}},\quad i = 1,2,3, \ldots ,N,\quad k > j$$

Therefore, if certain zero values of ${Y}_{i}$ is found between equal numbers of positive and negative values of ${Y}_{i}$, then the Sen’s Slope $Y$ is zero. The higher the number of equal values present in a time series, the higher the probability of a no-change trend for the data [57].

## Results

### Evaluation of model performance

The study compared the performance of a multi-model ensemble using both bias-corrected and raw data (Table 4). Initially, the raw multi-model ensemble performed poorly across selected stations in the Greater Accra region. For instance, NSE values for Prcp ranged from − 0.5 to 0.43, and R^2^ ranged from 0.04 to 0.58. Additionally, metrics like Pbias and RMSE indicated inadequate performance (Table 4). Similar results were observed for Tmin. However, the raw multi-model ensemble for Tmax performed well, particularly in virtual stations, even without bias correction.

**Table 4:** Model performance in terms of NSE, *R*^2^, Pbias, RMSE

	Station	Bias corrected				Raw			
		NSE	R^2^	Pbias	RMSE	NSE	R^2^	Pbias	RMSE
Precipitation	Accra	0.98	0.98	− 3.85	253.01	0.12	0.5	− 44.1	3483.44
	Pokuase	0.97	0.98	− 0.56	198.11	0.27	0.46	− 30.5	3143.01
	Tema	0.98	0.99	− 4.17	198.15	− 0.5	0.04	− 49.9	4493.4
	Virt1	0.97	0.98	− 0.21	158.27	0.42	0.58	− 25	2302.1
	Virt2	0.96	0.99	1.33	116.02	0.28	0.49	− 29.1	2566.81
	Virt3	0.97	0.99	1.25	120.09	0.33	0.49	− 25.1	2473.14
	Virt4	0.98	0.98	0.63	128.17	0.32	0.51	− 28.3	2505.19
	Virt5	0.97	0.98	0.60	130.44	0.36	0.53	− 26.1	2428.1
	Virt6	0.97	0.98	0.42	131.96	0.43	0.51	− 20.2	2371.22
Maximum temperature	Accra	0.99	0.99	0.09	0.11	0.24	0.84	3.7	1.29
	Pokuase	0.99	0.98	0.15	0.13	− 4.23	0.73	− 15.5	4.99
	Tema	0.99	0.99	0.08	0.21	0.29	0.78	− 4.7	1.84
	Virt1	0.98	0.98	0.13	0.27	0.83	0.90	0.8	0.9
	Virt2	0.98	0.98	0.19	0.28	0.83	0.87	− 0.4	0.91
	Virt3	0.98	0.98	0.27	0.28	0.83	0.88	− 0.7	0.9
	Virt4	0.98	0.98	0.44	0.29	0.78	0.88	− 1.6	1.03
	Virt5	0.98	0.98	0.29	0.27	0.83	0.89	− 0.6	0.9
	Virt6	0.98	0.98	0.82	0.30	− 1.61	0.87	5.7	1.76
Minimum temperature	Accra	0.99	0.99	0.05	0.08	− 4.4	0.43	− 9.4	2.61
	Pokuase	0.99	0.99	0.07	0.05	− 86.2	0.17	− 56.9	14.26
	Tema	0.99	0.99	0.04	0.07	0.01	0.16	− 1.9	1.52
	Virt1	0.97	0.97	0.11	0.06	0.02	0.51	− 4.4	1.63
	Virt2	0.97	0.97	0.17	0.26	0.28	0.36	− 1.7	1.38
	Virt3	0.97	0.97	0.14	0.28	0.16	0.32	− 2.6	1.49
	Virt4	0.96	0.96	0.18	0.16	0.07	0.44	− 3.6	1.57
	Virt5	0.95	0.96	0.15	0.19	0.21	0.56	− 2.7	1.21
	Virt6	0.95	0.96	0.12	0.17	− 9.99	0.89	− 10.9	2.96

Following the application of a distribution mapping algorithm, the performance of the multi-model ensemble improved (Table 4). For instance, NSE values for raw Prcp improved significantly, ranging from 0.96 to 0.98 after statistical bias correction. Similarly, R2 values for bias-corrected Prcp ranged between 0.98 and 0.99. Pbias and RMSE metrics also showed improved model performance post-bias-correction. Conversely, bias-corrected Tmax exhibited strong performance with NSE and R2 values ranging from 0.98 to 0.99, while bias-corrected Tmin ranged from 0.96 to 0.99 across selected stations (Table 4). However, both bias-corrected Tmax and Tmin exhibited variability in performance, with Pbias ranging from 0.04 to 0.82.

Overall, the multi-model ensemble's bias-corrected performance for Tmax, Tmin, and Prcp showed RMSE ranges of 0.11–0.30 °C, 0.05–0.28 °C, and 116.02–253.01 mm, respectively. The findings highlight discrepancies between overestimation and underestimation in the raw multi-model ensemble. Notably, simulated Tmax, Tmin, and precipitation demonstrated positive outcomes in terms of RMSE, NSE, R2, and Pbias (Table 4). Appendix 1 provides a graphical comparison between the multi-model ensemble and raw datasets.

### Quantifying historical and future drought events in Greater Accra

**Fig. 2:**
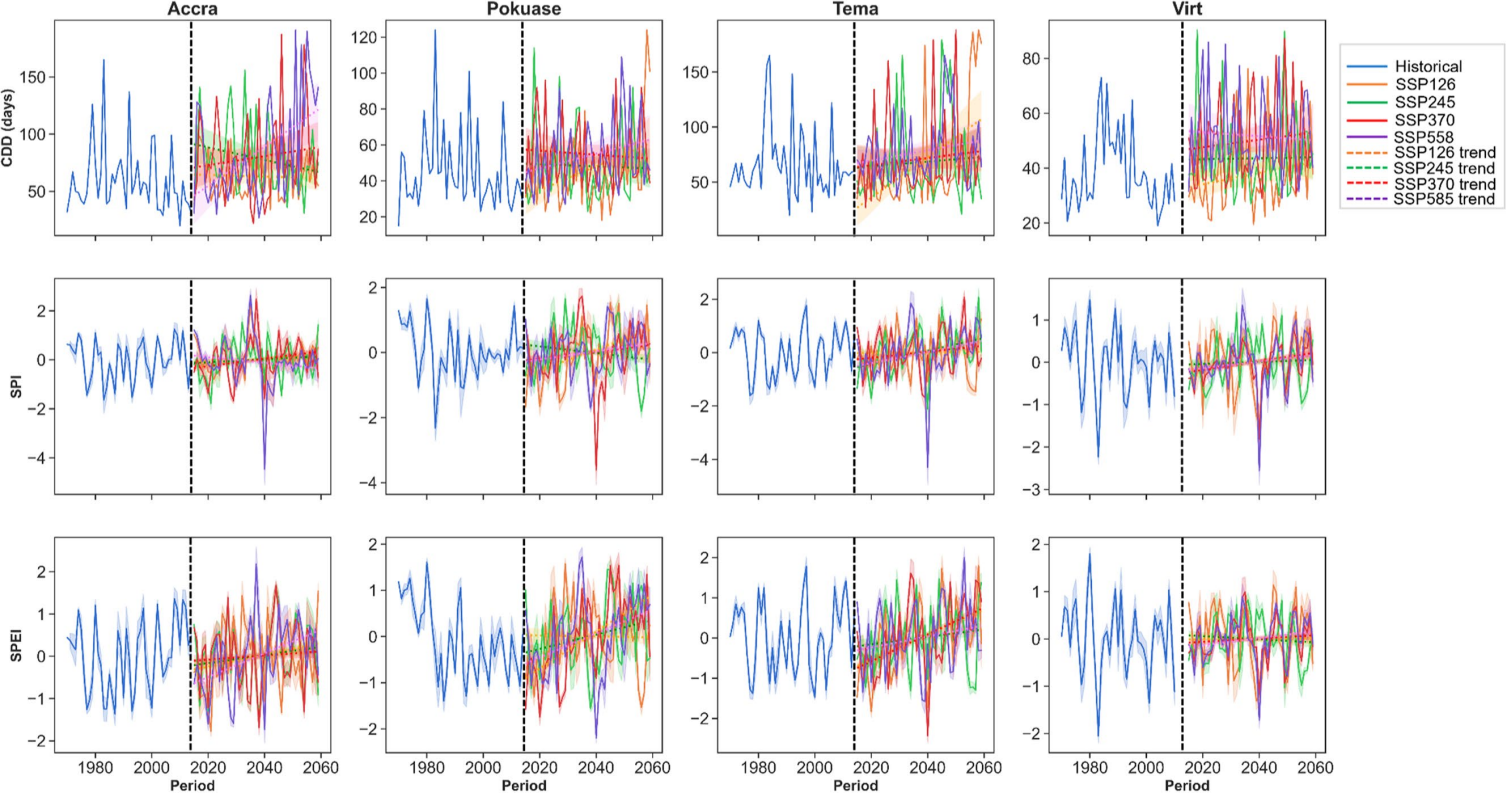
Temporal trends in annual mean CDD, SPI, and SPEI for the historical period, SSP126, SSP245, SSP370 and SSP585 scenarios between 1979 and 2059 in the Greater Accra region. The vertical black dash line separates the historical period from the future period

**Fig. 3:**
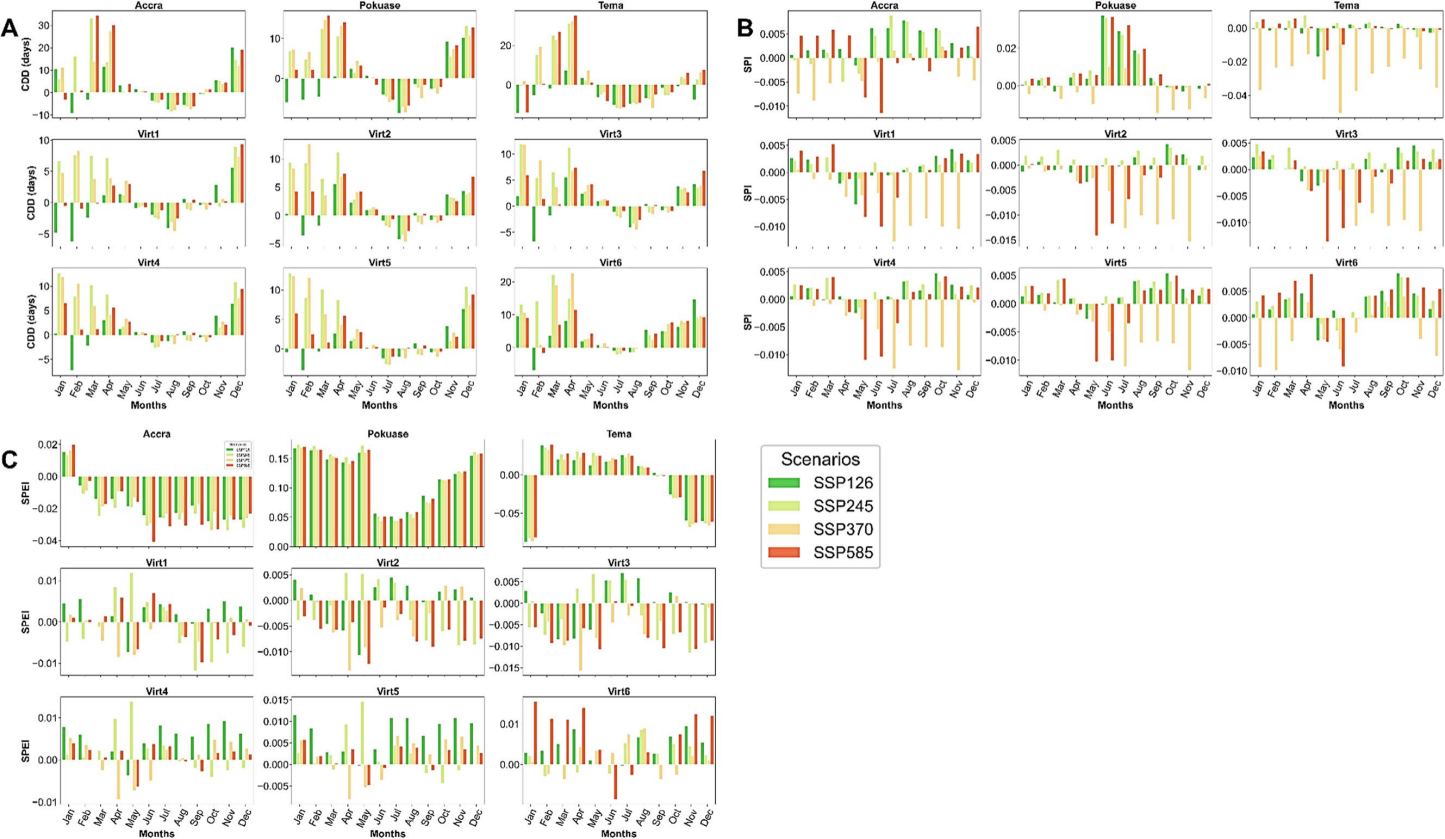
Mean monthly changes in CDD (**A**), SPI (**B**) and SPEI (**C**) under SSP126, SSP245, SSP370 and SSP585 scenarios relative to the historical period in the Greater Accra region

The temporal trend analysis of CDD indicates a declining pattern during the historical period across all selected stations (Fig. 2). However, under the SSP scenarios, especially SSP370 and SSP585, an increasing trend in CDD is anticipated across all stations in the region (Fig. 2). Accra and Tema are expected to experience higher CDD compared to other stations (Fig. 2). For instance, by 2059, CDD is projected to exceed 150 days under SSP370 and SSP585 at Accra, compared to less than 50 days in 2014 (Fig. 2). This signifies a substantial increase in CDD under SSP370 (Corrected Zc = 2.509, Sen’s slope = 0.259) and SSP585 (Corrected Zc = 3.627, Sen’s slope = 0.386) scenarios in Accra (Appendix 1). The mean monthly changes in CDD show a decrease in most months, reflecting seasonal patterns with higher CDD expected generally from November to April across all selected stations (Fig. 3). Specifically, March is anticipated to see the greatest increase in CDD under SSP585 at Accra, Pokuase and Tema (Fig. 3). Similar trends are expected across virtual stations (Fig. 3).

The SPI has shown an increase during the historical period in Accra (Corrected Zc = 1.99, Sen slope = 0.001), with no significant trend observed in Tema, while virtual stations indicated a decreasing trend (Appendix 1). However, under future scenarios, SPI is expected to decrease notably under SSP370 at Accra (Corrected Zc = − 3.821, Sen slope = − 0.001) and Pokuase (Corrected Zc = − 2.496, Sen slope = − 0.001). Similar decreases are anticipated under SSP585 at Accra, Pokuase, and Virtual Station 6 (Appendix 1), albeit with relatively weak trends based on Sen's slope coefficient. However, SPI is projected to drop significantly (− 4) by 2040 under SSP585 at Accra and Tema (Fig. 2), with extreme drops (up to − 2.5) expected across the region under SSP370 by 2040 (Fig. 2). Mean monthly changes reveal mixed signals across various months in Accra, with the most significant drop expected in June under SSP585 (Fig. 3). Generally, SPI is expected to decrease from October to March, with slight increases under certain scenarios (Fig. 3). At Tema, SPI is forecasted to decrease in all months, particularly under SSP585, with mixed trends expected at virtual stations (Fig. 3).

Regarding the SPEI, an increase was observed during the historical period in Accra (Corrected Zc = 1.755, Sen slope = 0.001) (see Appendix 1). Conversely, SPEI showed a general decrease across all stations during the historical period. However, under future scenarios, SPEI is expected to increase across most stations (excluding Virtual Station 6) under SSP585 (see Appendix 1). Yet, significant decreases (− 2) in SPEI are projected under SSP370 and SSP585 at Tema and Pokuase, respectively in the 2040s (Fig. 2). Mean monthly changes in SPEI (Fig. 3) indicate an expected decrease across all months except January in Accra by 2059. Conversely, at Pokuase, SPEI is anticipated to increase across all months, with February to August showing the most notable increases (Fig. 3). Similar mixed trends as in SPI are expected for future SPEI at virtual stations (Fig. 3).

**Fig. 4:**
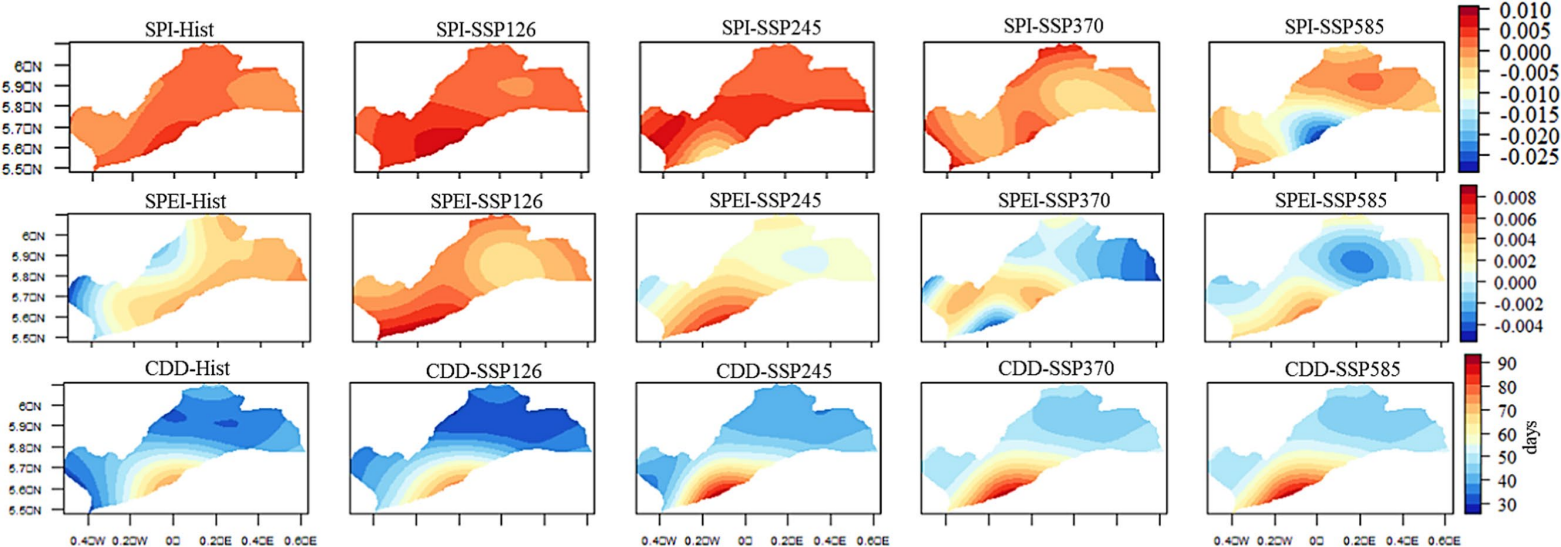
Historical and future annual mean CDD, SPI, and SPEI under the historical period, SSP126, SSP245, SSP370 and SSP585 scenarios between 1979 and 2059 in the Greater Accra region

The spatial distribution analysis of mean annual CDD and SPEI reveals heightened drought impacts under SSP245, SSP370, and SSP585 in the southwestern part of the region, encompassing Accra, Tema, and Pokuase (Fig. 4). For instance, CDD in Accra is projected to increase by up to 90 days under SSP245, SSP370, and SSP585, while SPI intensifies, particularly under SSP585 in Tema (Fig. 4). Additionally, SPEI is expected to shift from moderate to extreme drought conditions, especially under SSP370 and SSP585 in Accra, Tema, and Pokuase, compared to the historical period.

### Quantifying historical and future flood events in Greater Accra

**Fig. 5:**
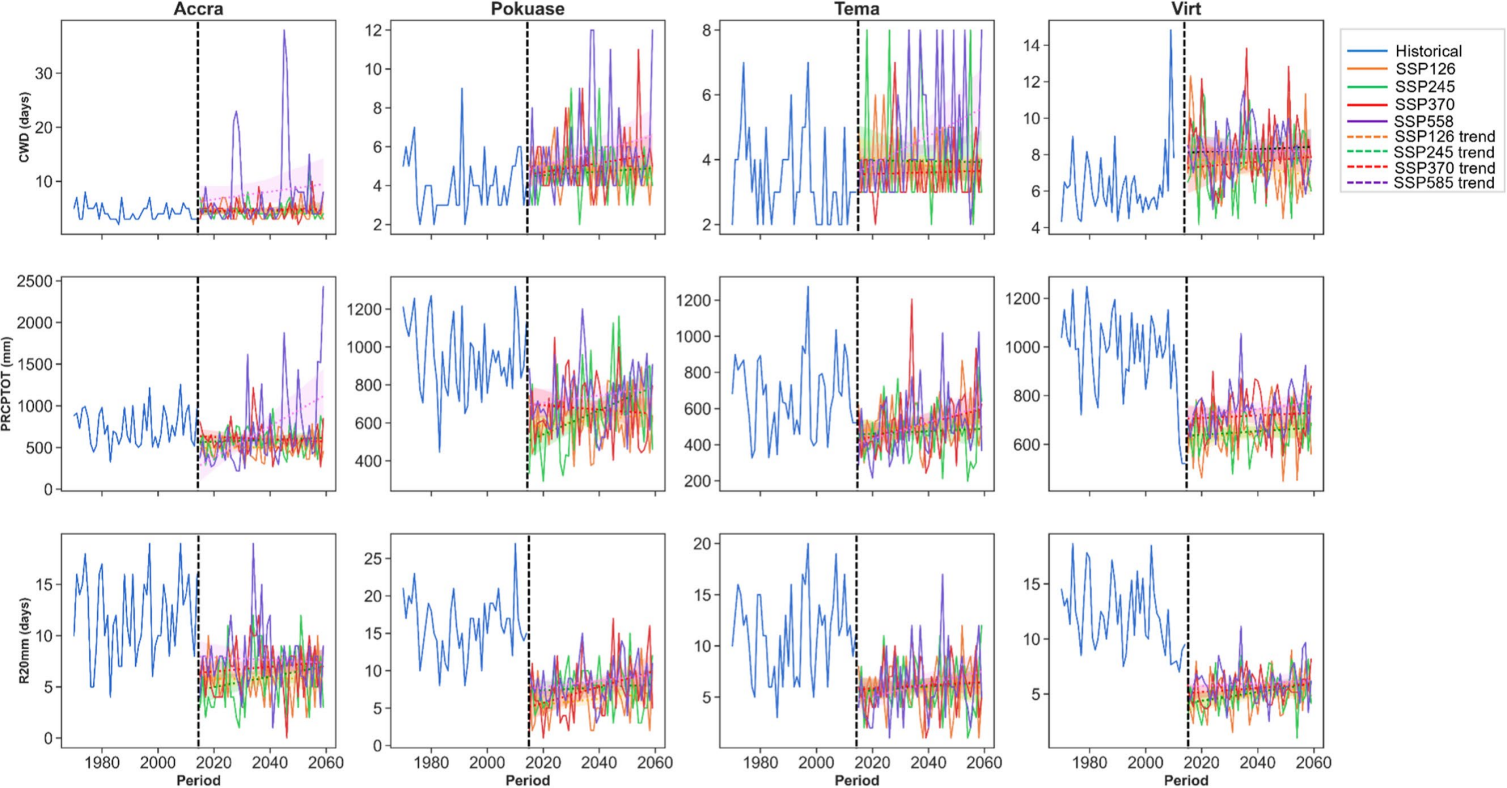
Temporal trends in annual mean CWD, PRCPTOT and R20mm for the historical period, SSP126, SSP245, SSP370 and SSP585 scenarios between 1979 and 2059 in the Greater Accra region. The vertical black dash line separates the historical period from the future period

**Fig. 6:**
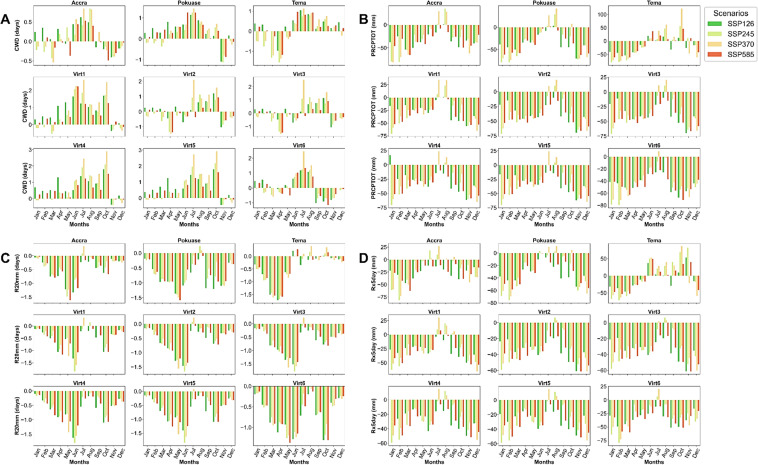
Mean monthly changes in CWD (**A**), PRCPTOT (**B**), R20mm (**C**) and Rx5day (**D**) under SSP126, SSP245, SSP370 and SSP585 scenarios relative to the historical period in the Greater Accra region

**Fig. 7:**
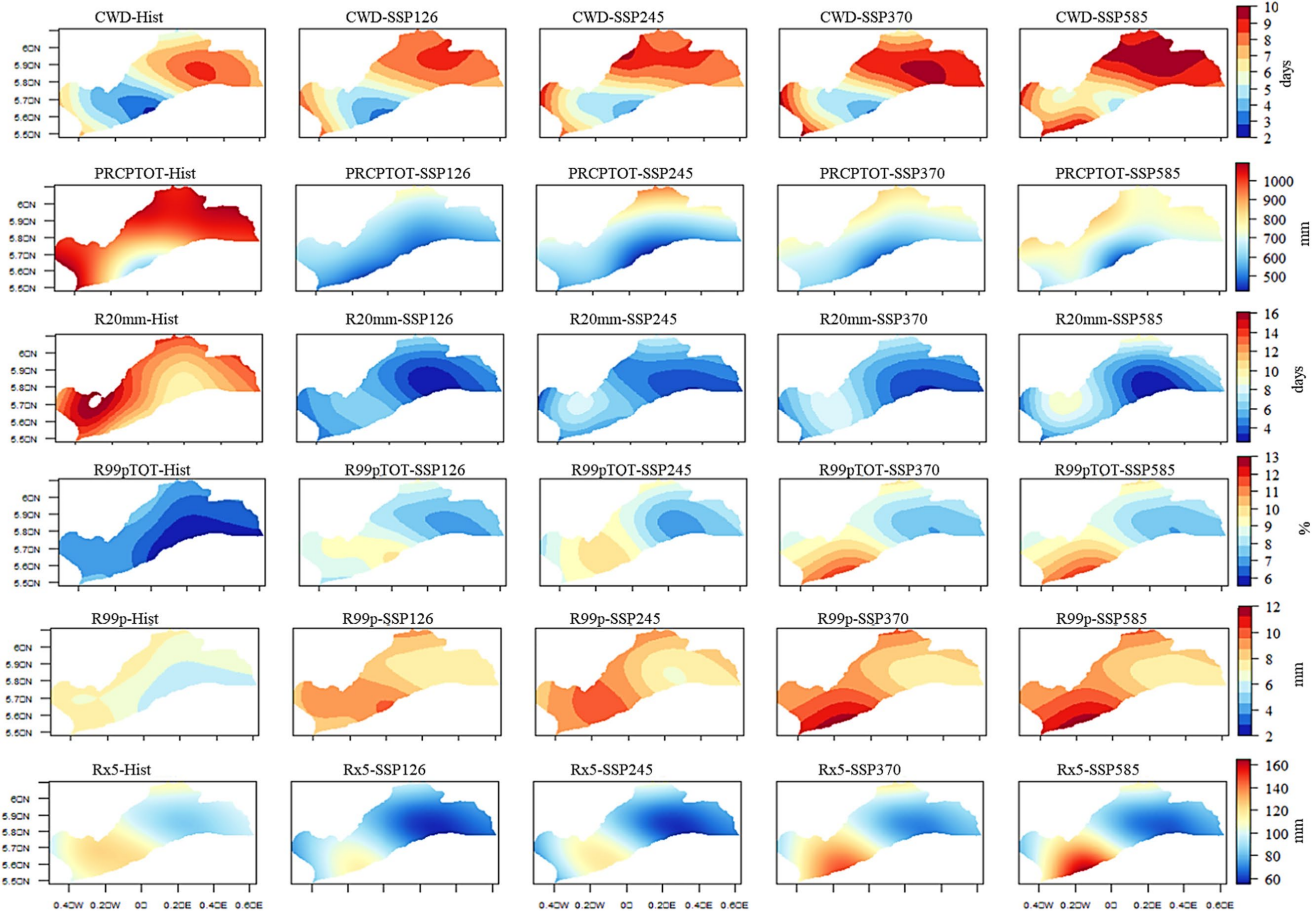
Historical and future annual mean CWD, PRCPTOT, R20mm, R99pTOT, R99, and Rx5days under the historical period, SSP126, SSP245, SSP370 and SSP585 scenarios between 1979 and 2059 in the Greater Accra region

In the historical period, the trend in CWD showed a decline in Accra and Tema stations, with an increase observed at the other stations, as indicated by corrected Zc values (see Appendix 1). However, Sen’s slope coefficients revealed no significant overall trend across all stations during this period (Appendix 1). Under SSP scenarios, a generally trendless pattern is expected. Nevertheless, marginal increases, particularly in Accra, Pokuase, and Tema, are evident under SSP370 and SSP585 (Fig. 5). Mean monthly changes in CWD did not exhibit significant variations under the SSP scenarios (Fig. 6). However, the mean annual CWD is projected to increase to approximately 10 days in the northeastern part of the study area under SSP370 and SSP585 (Fig. 7).

For PRCPTOT, a declining trend from the historical period to the future is expected across selected stations (Fig. 5). Historical data shows a declining trend in PRCPTOT across all stations except Accra, albeit at 5% significance level (Appendix 1). In the future, PRCPTOT is anticipated to increase at all stations except Pokuase (Appendix 1), particularly under SSP245 and SSP585. Accra, for example, is projected to record over 2000 mm of PRCPTOT under SSP585 in 2059 (see Fig. 5), indicating potential extreme events in these areas during specific periods. Mean annual PRCPTOT is expected to rise in the northeastern Greater Accra region under SSP245 and SSP370, while SSP585 forecasts an increase across Greater Accra (excluding Tema and virtual station 5) (Fig. 7). Mean monthly changes in PRCPTOT suggest decreases in all months except July and August at most stations, excluding Tema (Fig. 6).

Similarly, R20 mm is expected to decrease from the historical period to the future (Appendix 1). However, corrected Zc values indicate an expected increase in R20 mm under SSP126 and SSP245 across all stations, with statistically significant trends in some stations (Appendix 1). Pokuase and Tema are expected to experience a surge in R20 mm under SSP585 (Corrected Zc = 4.047 and 3.573 respectively) (Fig. 7). Mean annual R20 mm shows a general decrease under SSP scenarios, with intensified decreases in the southeastern part of Greater Accra (Fig. 7). Mean monthly changes in R20 mm reveal decreases in all months except July and August at Accra and Pokuase (Fig. 6), with similar trends expected across other stations except Tema (Fig. 6).

**Fig. 8:**
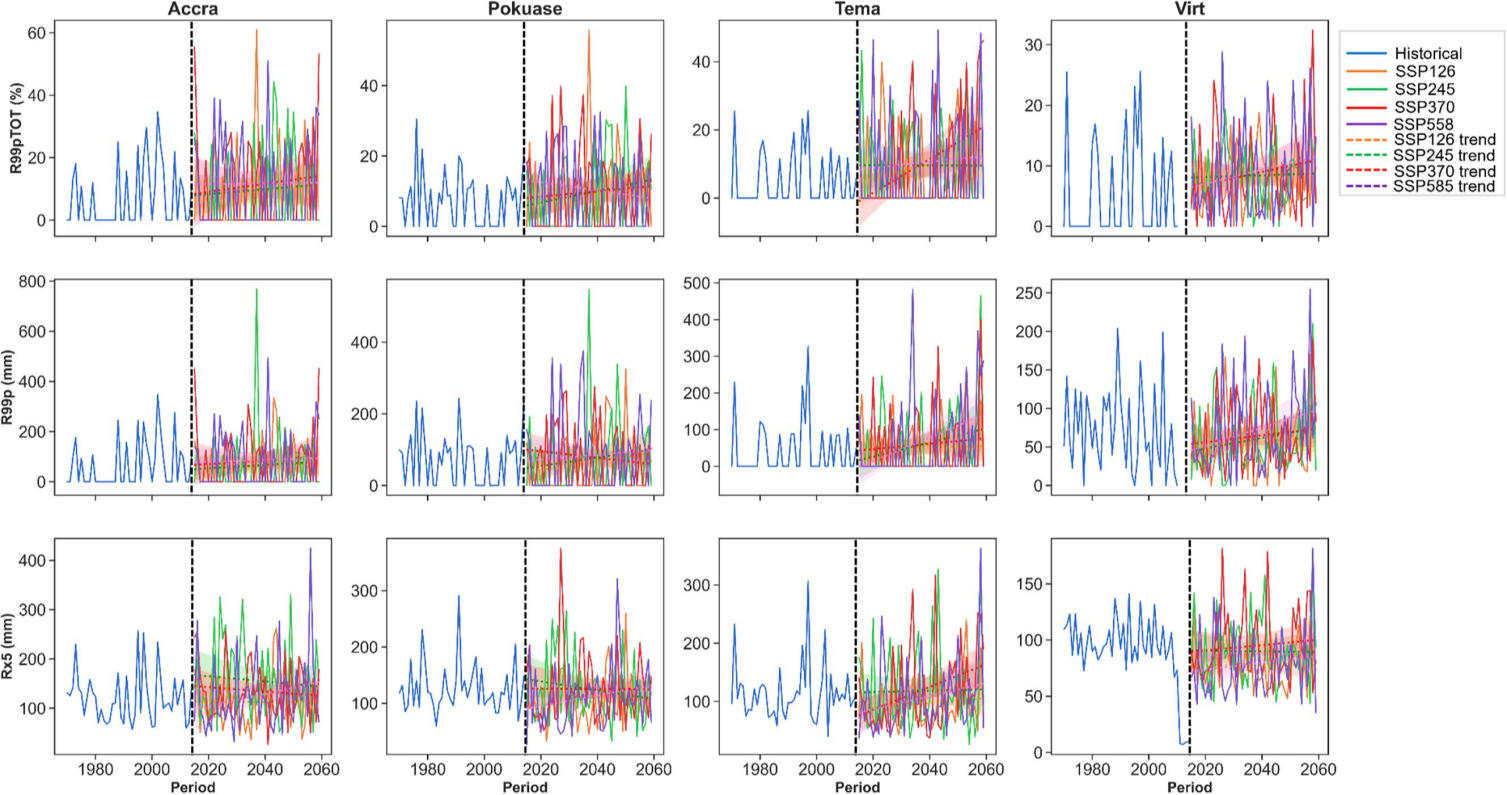
Temporal trends in annual mean R99pTOT, R99 and Rx5days for the historical period, SSP126, SSP245, SSP370 and SSP585 scenarios between 1979 and 2059 in the Greater Accra region. The vertical black dash line separates the historical period from the future period

The trend in R99pTOT showed an increase without significance at Accra, Pokuase, and Tema in the historical period, while virtual stations exhibited a significant decrease (Appendix 1). Under SSP126, SSP370, and SSP585 scenarios, R99pTOT is expected to increase at most stations, with significant increases at Tema under SSP126, SSP370, and SSP585 (Fig. 8). Mean annual R99pTOT indicates intensification at Tema and Accra (Fig. 8), with a projected 40% increase at Tema under SSP585 compared to 25% in the historical period (Fig. 8). Rx5days generally decreased in the historical period across stations (Appendix 1). However, under SSP370 and SSP585 scenarios, Rx5days are expected to increase significantly at most stations, including Accra (Fig. 8). The mean annual Rx5days is projected to intensify at Accra and Tema (Fig. 7), with mean monthly changes showing decreases in all months except June and July at Accra (Fig. 6). Pokuase and virtual stations generally exhibit decreases in all months except July and August, while Tema shows a drop from December to May, with the highest decrease in February under SSP245 (Fig. 6); however, an 85% increase is expected in October under SSP370 at Tema.

### Quantifying historical and future heatwave events in Greater Accra

**Fig. 9:**
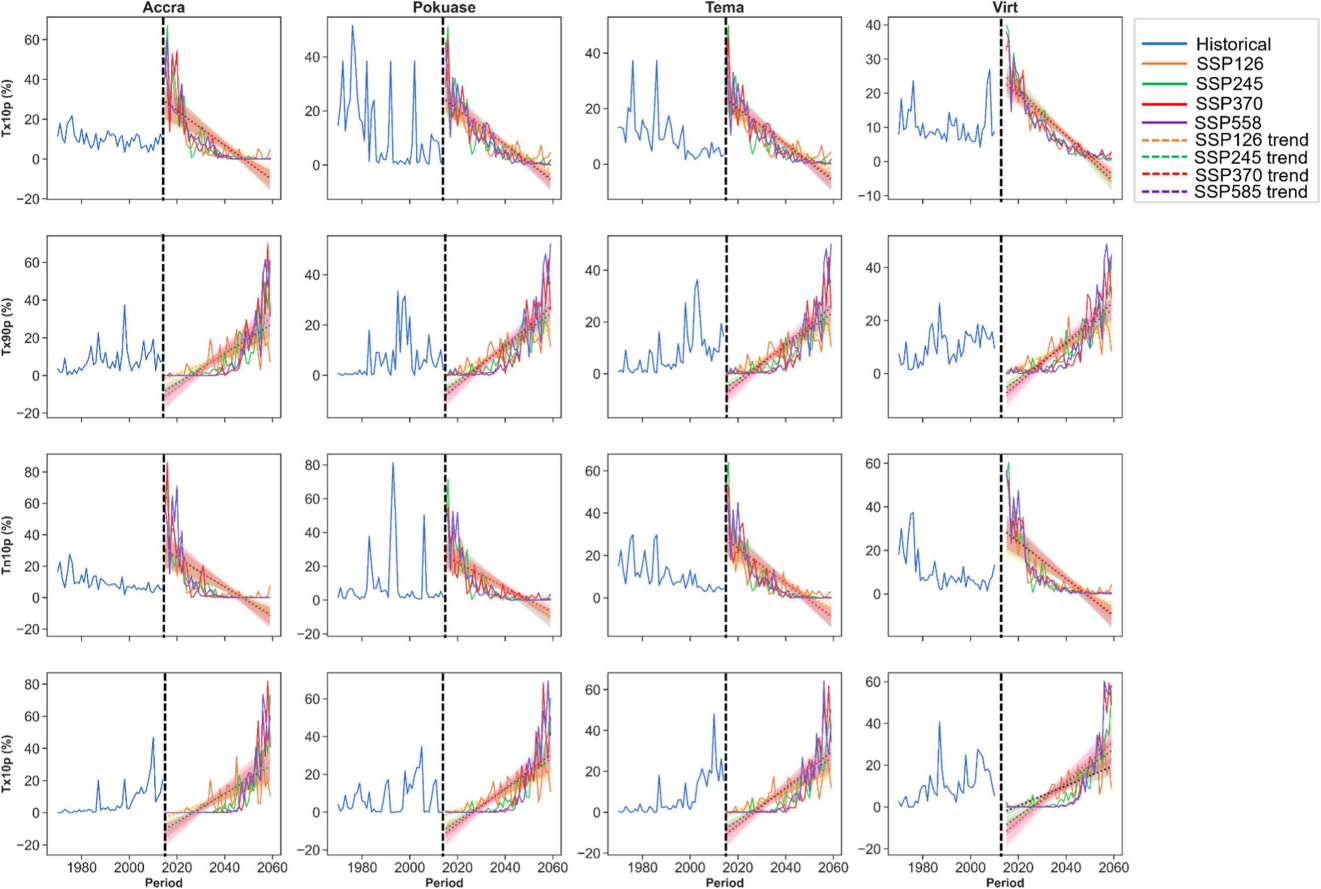
Temporal trends in annual mean Tx10p, Tx90p, Tn10p, and Tn90p for the historical period, SSP126, SSP245, SSP370 and SSP585 scenarios between 1979 and 2059 in the Greater Accra region. The vertical black dash line separates the historical period from the future period

**Fig. 10:**
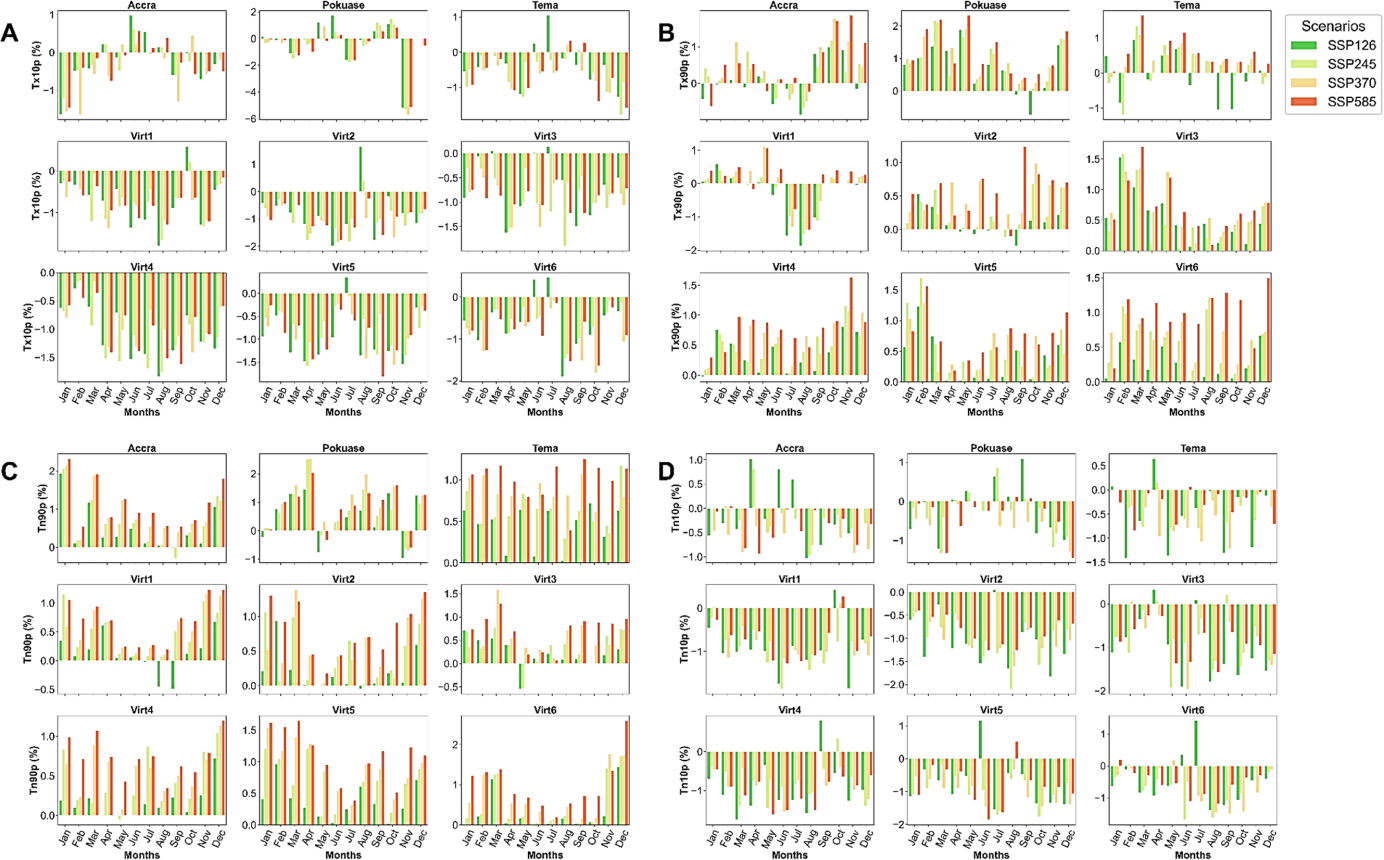
Mean monthly changes in Tx10p (**A**), Tx90p (**B**), Tn10p (**C**) and Tn90p (**D**) under SSP126, SSP245, SSP370 and SSP585 scenarios relative to the historical period in the Greater Accra region

Tx10p decreased across various stations in the region during the historical period. Significant declines were observed at Accra, Pokuase, Tema, and virtual stations at a 5% significance level (Appendix 1). Similar decreasing trends are expected under the SSP scenarios at the same significance level (Appendix 1). For example, Tx10p is projected to decrease from approximately 50% to nearly zero under SSP245, SSP370, and SSP585 at Accra compared to historical levels (Fig. 9). Similar decreases are anticipated at Pokuase, Tema, and virtual stations (Fig. 9). Mean monthly changes in Tx10p indicate expected drops in most months, particularly from January to March at Accra (Fig. 10). Similar trends are forecasted at Pokuase, with some increases in May and June under SSP126 (Fig. 10). Virtual stations show a general decline in Tx10p across almost all months under the SSP scenarios, with occasional spikes under SSP126.

Similarly, Tn10p exhibited a decreasing trend from the historical period into the future under SSP scenarios at a 5% significance level (Appendix 1). Mean monthly changes in Tn10p show declines under SSP scenarios across various stations in the region (Fig. 10).

Conversely, both Tx90p and Tn90p show an increase from the historical period into the future under SSP scenarios at a 5% significance level (Appendix 1). Tx90p showed a gradual increase historically, intensifying further under SSP370 and SSP585 across stations (Fig. 9). Mean monthly changes in Tx90p indicate increases in most months under SSP scenarios at Accra (Fig. 10), with intensified increases under SSP370 and SSP585 (Fig. 10). Pokuase is also expected to experience increases across almost all months, with the highest rate of change of about 2.3% under SSP585 (Fig. 10). Similar trends are expected across stations for Tn90p under SSP scenarios (Fig. 9). Mean monthly changes in Tn90p show increases across most months at various stations (Fig. 10), intensifying under SSP370 and SSP585.

**Fig. 11:**
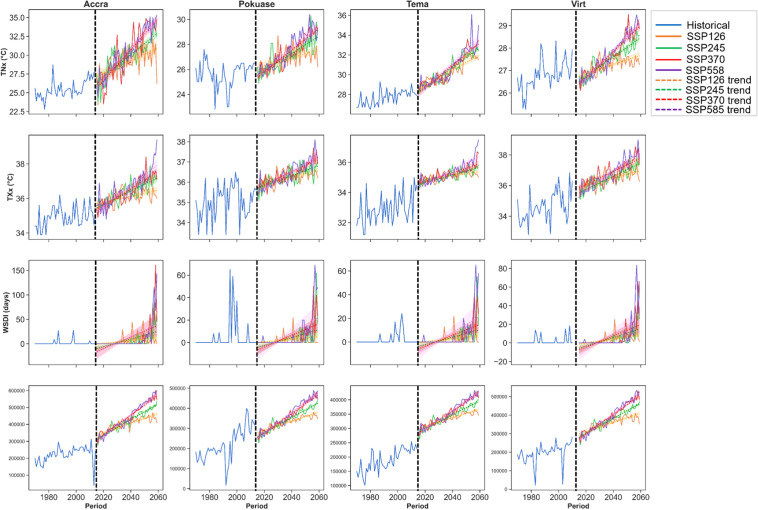
Temporal trends in annual mean TNx, TXx, WSDI, and CDDcold for baseline, SSP126, SSP245, SSP370 and SSP585 scenarios between 1979 and 2059. The vertical black dash line separates the historical period from the future period

**Fig. 12:**
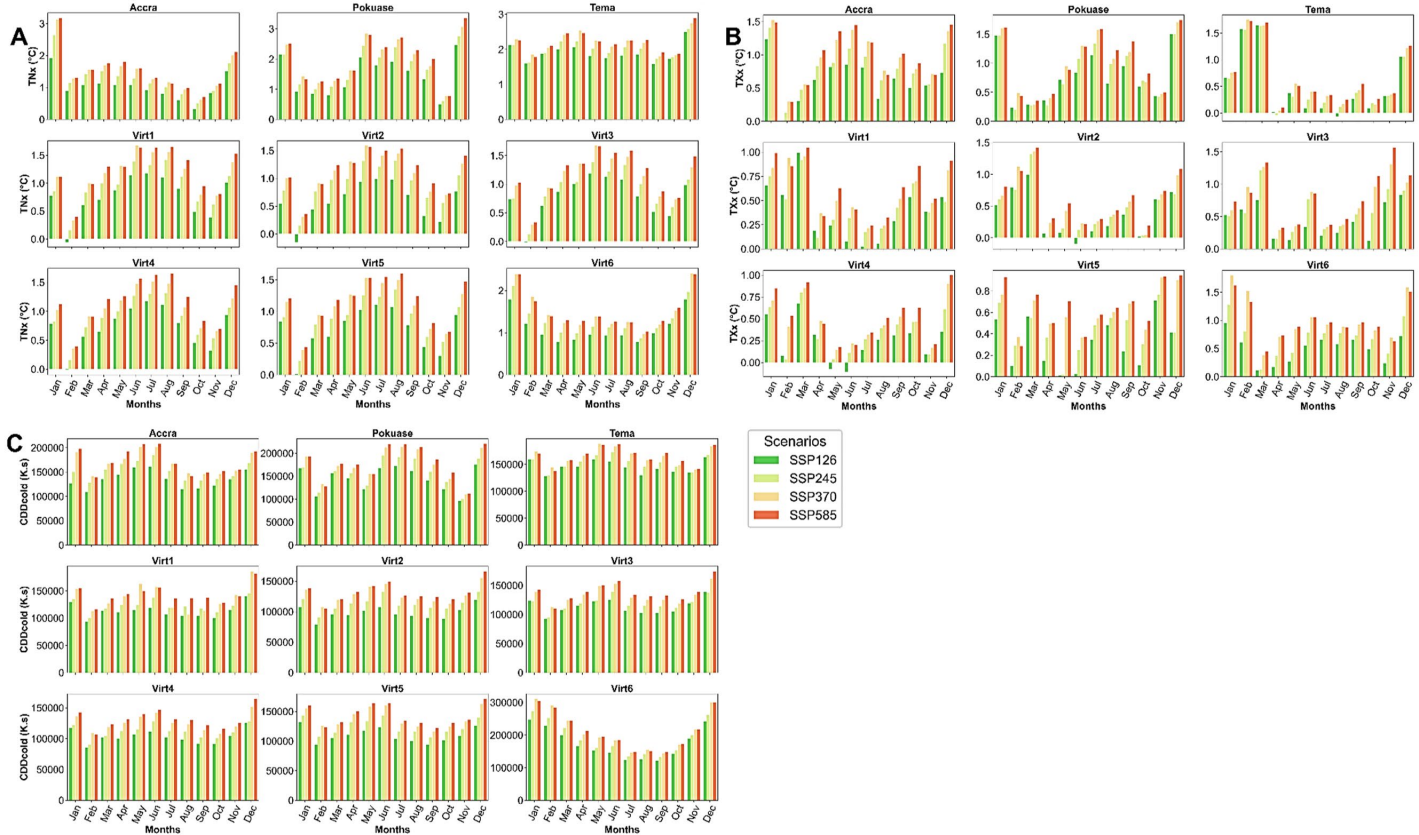
Mean monthly changes in TNx (**A**), TXx (**B**), and CDDcold (**C**) under SSP126, SSP245, SSP370 and SSP585 scenarios relative to the historical period in the Greater Accra region

TNx and TXx are anticipated to rise in both historical and future periods under SSP scenarios across all stations at a 5% significance level (Appendix 1). For example, TNx ranged between 22 and 26 °C historically at Accra from 1979 to 2014. This is projected to increase to about 40 °C under SSP370 and SSP585 from 2015 to 2059 (Fig. 11). Similar increases are expected at Pokuase, Tema, and virtual stations (Fig. 11). Mean monthly changes in TNx show a general increase across all stations and SSP scenarios. However, TNx is expected to intensify under SSP245, SSP370, and SSP585, with January expected to see the highest rate of change of about 3.2 °C under SSP585, making it the hottest month followed by December at Accra (Fig. 12). Similar trends are anticipated at other stations, with Tema projected to be the hottest station at night (Fig. 12). TNx under SSP126 is generally expected to show less severe changes compared to other scenarios (Fig. 10). Similarly, TXx is projected to increase from 1979 to 2059 across all stations under different SSP scenarios at a 5% significance level (Appendix 1). For instance, TXx is expected to reach approximately 40 °C and 38 °C at Accra and Pokuase, respectively, compared to historical values of 36 °C and 37 °C, respectively (Fig. 11). While TXx is less severe under SSP126, it intensifies under other scenarios. Mean monthly changes in TXx indicate January as the hottest month, with a rate of change of about 1.58 °C under SSP585 at Accra (Fig. 12). Similar patterns are expected at Pokuase, Tema, and other stations (Fig. 12).

CDDcold shows statistically significant patterns in both historical and future periods across all stations (refer to Appendix 1). For example, CDDcold is projected to surge to about 600,000 degree-days under SSP scenarios, compared to approximately 300,000 degree-days in the historical period at Accra (Fig. 11). Similar increases are expected at Pokuase and Tema (Fig. 11). Mean monthly changes in CDDcold indicate increases across all months, with January expected to be the hottest month at Accra (Fig. 12). Similar increases are expected at Pokuase, Tema, and virtual stations (Fig. 12), with December and January likely to see the highest rise in CDDcold across all stations under SSP scenarios (Fig. 12).

**Fig. 13:**
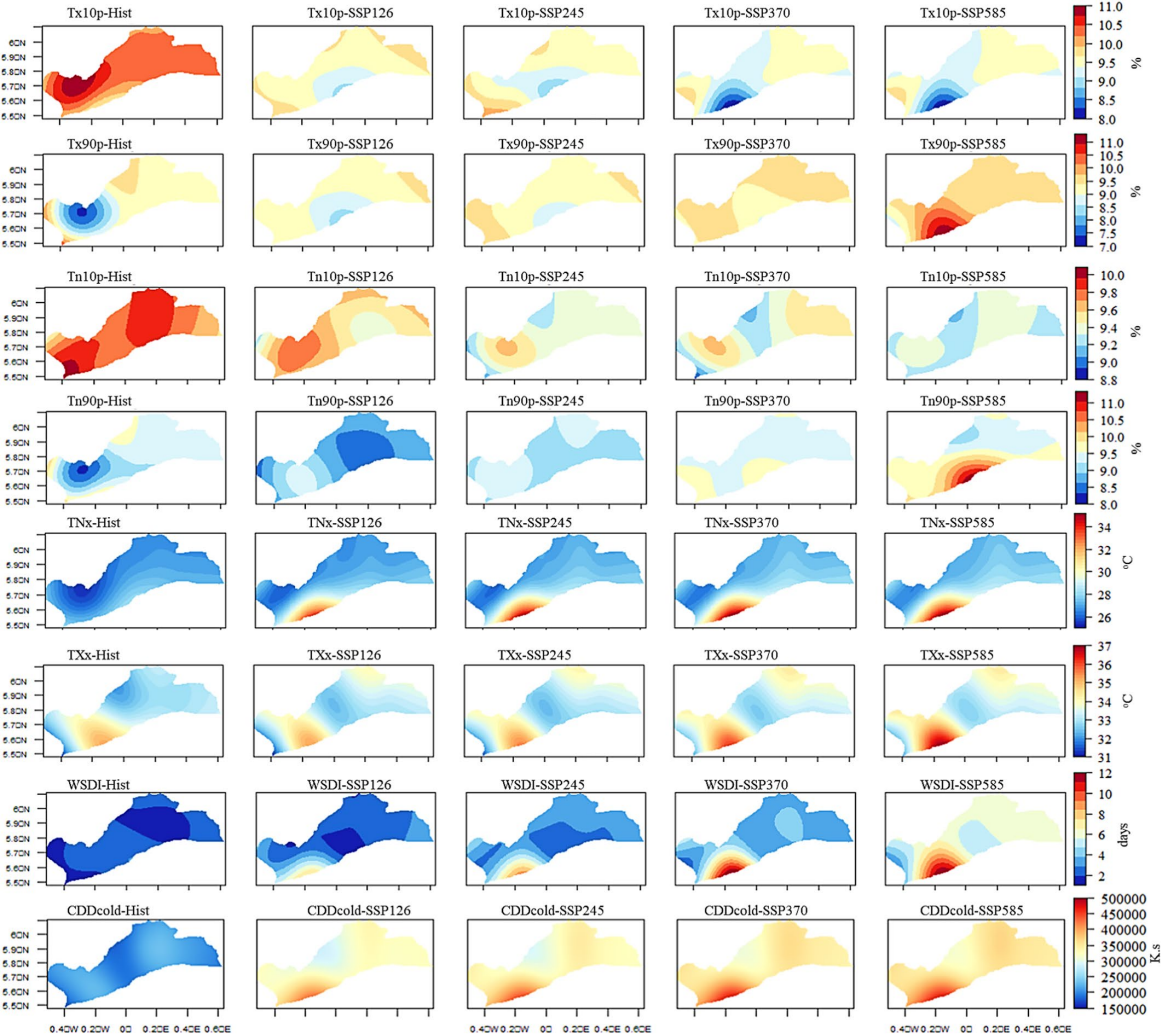
Historical and future annual mean Tx10p, Tx90p, Tn10p, Tn90p, TNx, TXx, WSDI, and CDDcold under the historical period, SSP126, SSP245, SSP370 and SSP585 scenarios between 1979 and 2059 in the Greater Accra region

Overall, heatwave indices are projected to intensify in the future, particularly under SSP245, SSP370, and SSP585 as shown by the spatial distribution (Fig. 13). Mean annual Tx10p is expected to range between 8–11% across Greater Accra from 1979 to 2059 (Fig. 13). While Tx10p increased historically, especially at Pokuase, a decline is expected under SSP scenarios, dropping to about 8% at Accra under SSP370 and SSP585 (Fig. 13). Tn10p increased in southwestern and northeastern parts historically from 1979 to 2014 (Fig. 12), but is expected to decrease to about 9.4% under SSP370 and SSP585, especially at Accra and Tema (Fig. 13). Mean annual Tx90p is projected to increase to about 11% under SSP585 at Accra, compared to 8% in SSP126 and historical periods (Fig. 13). Similarly, the mean annual Tn90p is expected to increase across the study area, particularly at Tema and Accra under SSP585 (Fig. 13). TNx and TXx are anticipated to record their highest annual means at Accra under SSP370 and SSP585 by 2059, reaching about 35 °C and 37 °C, respectively, compared to historical averages of 26 °C and 31 °C (Fig. 13). Mean annual WSDI and CCDcold are also expected to intensify at Accra under SSP370 and SSP585 (Fig. 13).

## Discussion

### Model performance evaluation

The multi-model ensemble demonstrated strong performance across various metrics in the study area. The results are consistent with recent studies in Ghana and other West African countries using a similar number of CMIP6 GCMs. For instance, Jacob et al. [58] reported R^2^ values ranging from 97 to 99 for Prcp, Tmax, and Tmin, with Pbias ranging from − 3.8 to 0.4, 0.04 to 0.3, and 0.04 to 0.2 respectively. RMSE values varied from 180 to 250 mm, 0.01 to 0.04, and 0.01 to 0.06 for Prcp, Tmax, and Tmin respectively. Similarly, Kouman et al. [59] observed comparable multi-model ensemble performance across various metrics.

Notably, the CMIP6 models, characterized by high resolution, exhibit enhanced accuracy, reduced precipitation error, and improved correlation compared to CMIP5 [60]. The performance of Tmax and Tmin in the multi-model ensemble generally surpassed that of Prcp, aligning with previous findings [58, 59]. This disparity can be attributed to the lower variability of temperature compared to precipitation, thereby enabling more accurate predictions. The conditional nature of precipitation, influenced by factors such as local and regional-scale predictors like atmospheric pressure and humidity, poses challenges for models to achieve precise predictions [61, 62]. However, RMSE and Pbias metrics reveal varying levels of uncertainty across the stations. Previous studies have noted similar uncertainties following the application of distribution mapping and linear scaling bias-correction methods [39, 58, 59].

### Impacts of historical and future drought events in Greater Accra

In the historical context, while CDD displayed a marginal decreasing trend, SPI, and SPEI indicated a marginal increasing trend, suggesting a generally weak drought intensity until 2014. This implies that the Greater Accra region was wetter during the historical period, aligning with the findings of [63], which indicated an increasing SPI trend along the coast of Ghana, particularly in areas such as Accra, Tema, Axim, and Saltpond. However, historical drought patterns exhibited mixed trends with weak intensities, according to [63]. In contrast, the trends projected under the SSP scenarios indicate a departure from historical trends, with CDD, SPI, and SPEI expected to intensify, especially under SSP370 and SSP585 scenarios between 2020 and 2059 in the Greater Accra region. The spikes observed in the future drought indices reveal the likelihood of flash drought events in the Greater Accra region, given the expectation of short but intense droughts daily. According to Zhang et al. [64], the frequency of flash floods is projected to increase, particularly under higher emission scenarios, with a growth rate approximately 1.3 times higher than that under medium emission scenarios.

Accra and Tema are projected to be severely affected, suggesting that future socioeconomic activities and associated emissions in these cities may contribute to increased drought intensity, particularly under SSP370 and SSP585. For example, Olaoluwa et al. [65] found that human activities influencing precipitation (Prcp) and temperature have led to a medium level of confidence regarding the anthropogenic impact on observed changes in drought conditions.

Moreover, the SPI and SPEI under the SSPs are expected to vary, with the variations in moisture levels likely attributable to land–ocean–atmosphere linkages, topography, and landscape dynamics. Additionally, the monsoon winds in West Africa and the seasonal movements of the Intertropical Discontinuity (ITD) may influence these variations, as noted by [63]. The shift of the ITD from north to south based on seasons may trigger drought, while the reverse may result in wet conditions. Unusual sea surface temperature and associated large-scale teleconnection factors, including El Niño Southern Oscillation (ENSO) and the North Atlantic Oscillation (NAO), may also play a role in triggering extreme events in West Africa, particularly in Ghana [63]. Ankrah et al. [66] found that drought along the coast of Ghana has short- and long-term variations, with extreme drought events being higher in the Central and Greater Accra regions compared to the Volta region. Therefore, future climate change under the SSP scenarios is expected to increase the frequency and severity of droughts in the Greater Accra region, leading to potential water shortages, crop failures, and other impacts on the region's economy and society as well as agricultural activities, cultivated grasslands and barren surfaces [67].

Several challenges complicate the projection of drought conditions. For example, inconsistencies in data and the availability of reliable evidence make it difficult to correlate observed changes in drought conditions at a regional level [65]. Currently, the confidence level regarding projected increases in the duration and intensity of drought in certain regions of the world ranges from low to medium. This uncertainty stems from definitional ambiguities and issues with data availability [65]. Moreover, challenges persist in modeling all factors influencing drought occurrence, uncertainties related to the impact of climate drivers like El Niño on drought, and changes in land–atmosphere feedback associated with drought [65].

### Impacts of historical and future flood events in Greater Accra

The trend of flood indices has shown a marginal increase during the historical period and is anticipated to intensify under the SSP scenarios. However, the future trend is not uniformly distributed across the Greater Accra region. While some stations are projected to experience a rise in the frequency, duration, and intensity of floods, other areas may observe minimal or no significant change in trend. This aligns with existing studies indicating that the dynamics of extreme precipitation events will exhibit non-uniform patterns in the future [34, 38, 61, 68, 69]. However, flood indices are anticipated to intensify more significantly under SSP585 in comparison to SSP126 and SSP245 [70]. The projected flood indices are likely to be severe due to the intensity and duration of some daily extreme precipitation indices. For example, the annual means of daily CWD, R99pTOT, R99p, and Rx5days are expected to increase in certain parts of the region compared to the historical period. This implies an increase in flooding in the future due to the heightened intensity and duration of flood indices in the region. However, the annual mean of daily PRCPTOT and R20 mm is expected to generally decrease between 2020 and 2059. Despite the expectation of low frequencies, particularly under SSP245, SSP370, and SSP585 scenarios, the projected rise in the intensity and duration of flood indices, combined with other factors such as urban sprawl, population growth, poor waste management, and construction in waterways (which is on the rise in the region), may lead to an escalation of flooding in the area. However, the IPCC report [71] indicates low to medium confidence in the observed climate-induced changes in the magnitude and frequency of floods at a regional level. This uncertainty stems from limitations in spatiotemporal data availability, insufficient evidence, and the complicating influences of land use and engineering practices.

Several studies attribute floods in the Greater Accra region to non-meteorological factors. For instance, Ansah et al. [13] noted that non-meteorological factors such as poor drainage systems, improper refuse disposal, construction on waterways, limited urban rooftop rain harvesting systems, limited tree planting, and intensive unplanned anthropogenic structures in flood-vulnerable zones play a significant role in contributing to the flood risk in Ghana, especially in major areas in Accra. Consequently, even mild downpours currently lead to some form of flooding in parts of the Greater Accra region [72]. However, there is low confidence in the evidence linking anthropogenic impacts to changes in the magnitude and frequency of flood occurrences. Similarly, confidence in projected flood changes is low due to insufficient evidence. However, there is medium confidence that rain-fed floods may increase in some regions due to the projected rise in heavy rainfall events [71].

### Impacts of historical and future heatwave events in Greater Accra

In general, heatwave indices in Greater Accra are anticipated to gradually rise in the historical period and intensify under the SSPs. A reduction in cool days is expected, signifying a probable increase in diurnal temperatures between 2020 and 2059, especially in Accra, where daytime temperatures are projected to surge, particularly under SSP245, SSP370, and SSP585. The study of [3] indicates that the hottest day's temperature rise is expected to be most pronounced in middle latitudes and semi-arid regions, surpassing the global warming rate.

Concurrently, as cold nights (Tn10p) decrease, warm nights (Tn90p) are forecasted to increase, particularly under SSP245, SSP370, and SSP585 at Accra and Tema. This suggests an overall intensification of daytime and nighttime temperatures between 2020 and 2059 under the SSPs, aligning with IPCC findings that warm indices have risen while cold indices, for both nights and days, have decreased. Nevertheless, nighttime temperatures are expected to generally surpass daytime temperatures in the region. This aligns with the findings of [16] that a more rapid rise in nighttime temperatures than daytime temperatures, resulting in a reduced diurnal temperature range in Greater Accra. Increased warming is foreseen, accentuating climate extremes, especially with expanding urbanization. Additionally, while Accra is projected to be warmer during the day, Tema is expected to experience warmer nights, likely due to daytime congestion and emissions in Accra, with people returning home at night, contributing to warmer nights in Tema and an urban heat island effect in the region.

Indicators like TNx, TXx, Warm Spell Duration Index (WSDI), and Cooling Degree Days for cold temperatures (CDDcold) are expected to also increase. The consequences of heatwave indices extend to various detrimental impacts on human health and society. For example, prolonged extreme diurnal temperatures exceeding 35 °C for hot days may result in health impacts. For example, warmer nights and days are projected to increase under SSP245, SSP370, and SSP585, potentially increasing population exposure to future heatwaves in the region [73, 74].

Moreover, extreme temperatures, both hot and cold, significantly affect electricity consumption in Greater Accra. Increased demand for air conditioning and heating due to extreme temperatures leads to elevated electricity consumption, impacting the region's power infrastructure.

For instance, studies by [75, 76] highlight that extreme temperatures correlate with increased residential electricity demand in Greater Accra. Extreme temperatures can affect power stations, reducing efficiency and output capacity, potentially causing power outages. The region's dependence on water-based cooling systems for power stations may face challenges due to extreme heatwave events and reduced water availability, impacting their operation. The results suggest a significant increase in heatwave events between 2020-and 2059 under the SSPs, particularly under the SSP585 scenario, indicating a potential rise in cooling demands for residential and industrial areas and potential strain on power infrastructure in Greater Accra.

In general, heatwave indices were found to be responsive to the radiative forcing of the SSP scenarios, showing a direct relationship between the heatwave indices and the SSP scenarios. Therefore, higher emission scenarios are associated with greater warming magnitudes in the region.

### Historical and future trends of extreme climate events over West Africa

Table 5 presents an overview of the historical and future trends in extreme indices across West Africa. The study reveals a notable increase in PRCPTOT during the historical period along the Guinean coast (Ghana), with consistent findings in other studies [66, 77] in Ghana. However, conflicting results emerge from existing studies [5, 78] over West Africa, showing a decreasing trend. For example, Diatta et al. [79] found a mixed trend, with decreasing PRCPTOT along the Guinean coast and increasing trends in areas like Western Mali and Northern Burkina Faso. Future projections vary by country, indicating a significant decrease in PRCPTOT along the Guinean coast, in line with the projections of [25]. Conversely, Abiodun et al. [46] project a significant increase in Nigeria (Lagos).

**Table 5:**
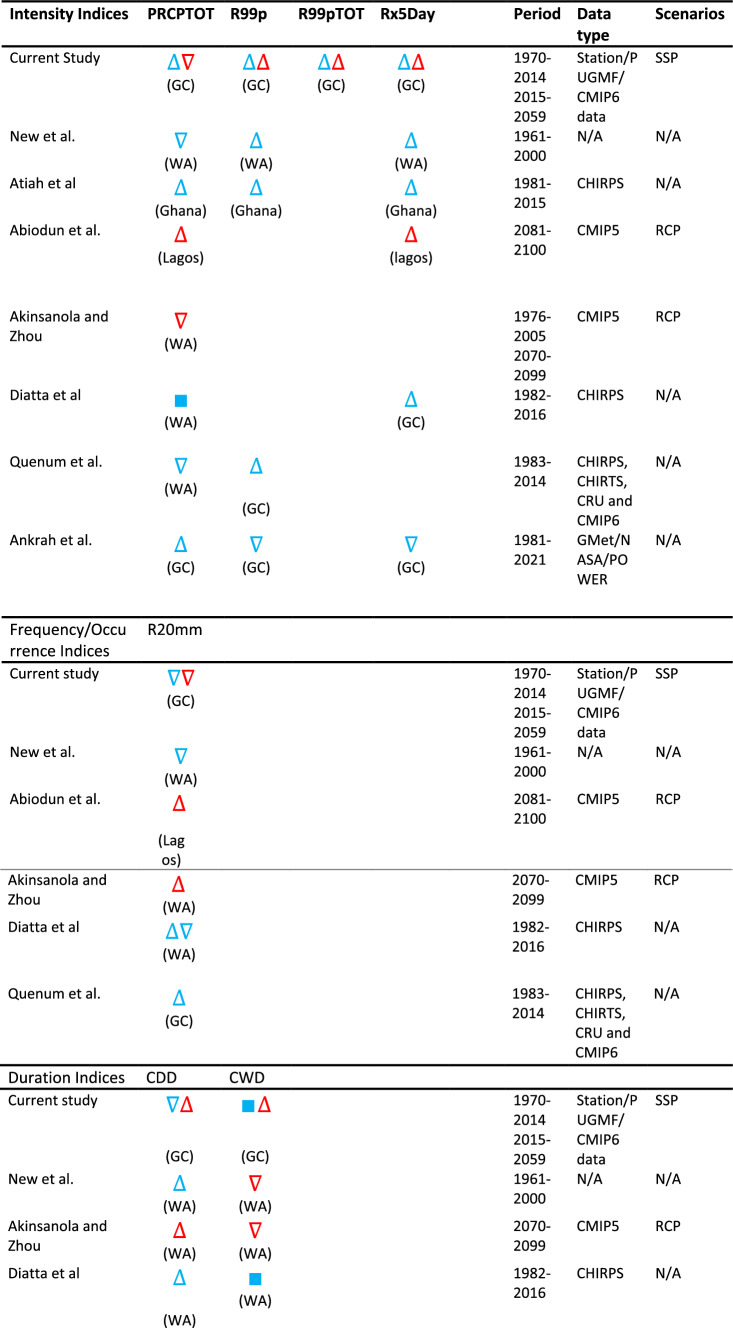

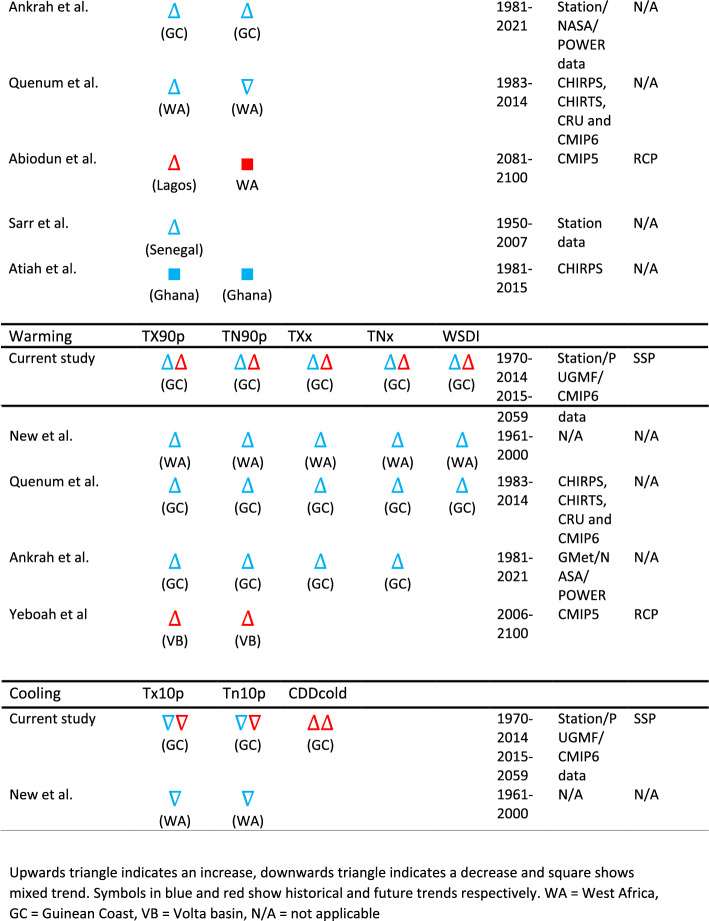
Trends of extreme climate indices over West African countries

Upwards triangle indicates an increase, downwards triangle indicates a decrease and square shows mixed trend. Symbols in blue and red show historical and future trends respectively

WA, West Africa; GC, Guinean Coast; VB, Volta basin; N/A, not applicable

The historical trend of very heavy precipitation (R99p) showed an increasing pattern over West Africa [5, 77], consistent with the current study's findings. Future projections suggest a general increase, particularly along the Guinean coast, in R99p, R99pTOT, and Rx5days (Table 4), indicating a higher risk of flooding in West Africa. Overall, rainfall intensity is expected to rise by the end of the twenty-first century. In contrast, the current study observed a decreasing historical trend in the amount of rainfall exceeding 20 mm (R20 mm), aligning with the overall trend in West Africa [5]. Future projections also indicate a decline. However, [79] found decreasing R20 mm along the Guinean coast and increasing trends in the whole Sahel region. Abiodun et al. [46] project an increase in Nigeria (Lagos), showing an erratic R20 mm trend over West Africa.

Concerning duration indices (Table 5), the current study found a marginal drop in CDD during the historical period, with future projections indicating an increase, especially along the Guinean coast. Previous studies show mixed trends in historical CDD, with an overall increasing trend over West Africa. For instance, Abiodun et al. [46] project an increasing trend in CDD in Nigeria (Lagos). The historical trend of CWD was mixed, with marginal increases in the Western Sahel and moderate drops in the eastern Guinean coast. Future trends suggest a marginal increase in CWD, varying across studies. Abiodun et al. [46] found a mixed trend, while Akinsanola and Zhou [25] project a decrease in West Africa. The intensification of precipitation extremes, coupled with changes in warm and cold indices, is expected to impact hydroelectric dams, water resources, and rain-fed agriculture in West Africa.

Examining warming and cold indices (Table 5), both previous and current studies indicate an increasing trend in warm indices and a decreasing trend in cold indices during the historical period, with intensification projected for the future over West Africa. Increased temperatures, especially in megacities, are anticipated by the end of the twenty-first century. Notably, projections from CMIP6 models align with CMIP5 models, reinforcing the confidence in these outcomes [3].

### Policy implications of the study

The Greater Accra region is anticipated to face brief but severe droughts, floods, and heatwaves under SSP245, SSP370, and the worst-case scenario (SSP585), although these impacts could be alleviated under the sustainable pathway of SSP126. It is crucial to integrate these indices into future climate policies for the region. Addressing Ghana's commitments under the Paris Agreement could potentially mitigate drought impacts from severe to moderate levels. However, even with global warming stabilized at 1.5 °C, regions like Ghana are projected to experience significant ecological and agricultural droughts [3]. Therefore, careful consideration of drought, flood, and heatwave indices under SSP245, SSP370, and SSP585 in the Greater Accra region is essential.

Disaster management plans (DMPs) must account for local and regional conditions of drought, flood, and heatwave severity, including timely monitoring of the natural environment with a focus on vegetation health and ecosystems [80], especially under SSP245, SSP370, and SSP585. An effective DMP should adopt a dynamic approach, continually preparing for and managing climate extremes [80]. This includes periodic assessments of precipitation, temperature impacts, and their implications for drought, flood, and heatwave events. Collaboration between districts and municipalities in the region is crucial to address climate extremes, ensuring accessibility of adaptation and mitigation strategies across relevant institutions.

Moreover, efforts should not only identify risks and vulnerabilities but also prioritize policy formulation, decision-making, implementation, and evaluation [80, 81]. Government and stakeholders must play proactive roles in mainstreaming adaptation and mitigation plans into effective policies. Proactive risk management policies should incorporate advanced measures, innovative tools, and scientific expertise [80]. Mainstreaming vulnerability considerations requires a coordinated framework involving policymakers, scientists, governments, and stakeholders at all levels of policy development. While challenges exist, overcoming them is feasible with dedication and appropriate structures [80].

## Conclusion

The observed and projected trajectory of Extreme Climate Events (ECEs) has become more critical for every country following the atrocities caused as a result. This study quantified the historical and future extreme climate over the Greater Accra region in comparison to other West African countries. The results of this study emphasize the pressing reality of climate change and its disproportionate impact on West African countries (notably megacities). As exemplified by the Greater Accra region, host to the bustling megacity of Accra, the vulnerability to extreme climate events (ECEs) such as floods, heatwaves, and droughts is a growing concern. The results of the study uncover the region's growing vulnerability to drought, flooding, and heatwave indices under the SSP scenarios (especially SSP245, SSP370, and SSP585). For instance, the study revealed a general increase in CDD under the SSP scenarios across the region. On the contrary, SPI and SPEI are expected to oscillate between negative and positive signals. Moreover, flood indices such as R99p, R99pTOT, Rx5days amongst others are expected to surge under the SSP245, SSP370, and SSP585 with a drop in PRCPTOT and R20 mm. Similar trends have been observed and projected over the West African region with the Guinean coast regions expected to be more vulnerable. The Greater Accra region is expected to be warmer during the day and night under the SSP245, SSP370, and SSP585 scenarios compared to SSP126 and the historical period. However, while Tema is expected to be warmer in the night, Accra is expected to be warmer in the day. Similarly, the West African subregion is expected to observe a sharp increase and decrease in warming and cooling indices respectively.

The insights derived from this study serve as a clarion call for collaborative climate action from various stakeholders and policymakers in Greater Accra as well as other West Africa regions to invest in climate-smart technologies designed for megacities to mitigate the adverse effects of future droughts, floods, and extreme temperatures. Additionally, efforts to reduce greenhouse gas emissions and slow the pace of climate change will be critical to protecting vulnerable communities and ecosystems from the impacts of drought, flood, heatwave, and other climate-related hazards in the future. As such considering these findings in climate-smart policy formulation and planning for future climate adaptation and mitigation strategies is of urgent need to contribute to the nationally determined contribution of the Paris Agreement and address the sustainable development goal 11 (Sustainable cities) and 13 (Climate action).

## Figures and Tables

**Fig. 14:**
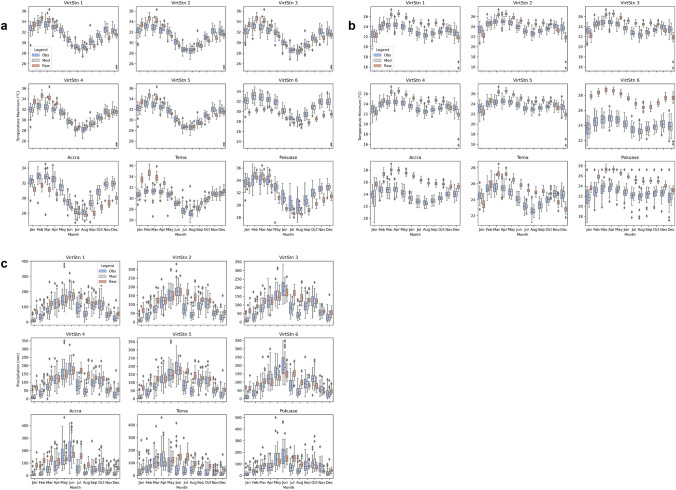
**a** Ensemble bias-corrected and raw Tmax comparison relative to the baseline periods (1979–2014). Where Obs and Mod represents observed and modeled respectively. **b** Ensemble bias-corrected and raw Tmin comparison relative to the baseline periods (1979–2014). Where Obs and Mod represents observed and modeled respectively. **c** Ensemble bias-corrected and raw prcp comparison relative to the baseline periods (1979–2014). Where Obs and Mod represents observed and modeled respectively

## Data Availability

Data will be made available on reasonable request.

## Appendix 1

See Table 6.

**Table 6:** Results of the Modified Mann–Kendall test for historical and future extreme climate projections under the SSP scenarios

Station	Historical		Future							
			SSP1		SSP2		SSP3		SSP5	
Index	Corrected Zc	Sen’s slope	Corrected Zc	Sen’s slope	Corrected Zc	Sen’s slope	Corrected Zc	Sen’s slope	Corrected Zc	Sen’s slope
*Ensemble CDD*										
Accra	− **2.613**	− 0.295	− 0.409	− 0.014	− 0.591	− 0.065	**2.509**	0.259	**3.627**	0.386
Pokuase	− 2.660	− 0.222	**2.700**	0.415	0.560	0.052	**3.514**	0.260	**3.554**	0.271
Tema	− 0.506	− 0.069	**2.558**	0.774	0.245	0.029	**2.193**	0.209	**2.002**	0.308
Virt1	0.293	0.055	**4.390**	0.200	**1.222**	13.208	0.092	0.002	− 0.53	− 0.045
Virt2	0.440	0.044	**3.099**	0.193	0.889	0.049	− 0.183	0.001	0.985	0.060
Virt3	− 0.511	− 0.061	**2.910**	0.182	0.900	0.048	0.161	0.012	1.071	0.066
Virt4	− 1.586	− 0.200	**3.867**	0.186	**1.717**	0.094	**2.196**	0.133	1.136	0.054
Virt5	− **1.930**	− 0.221	**5.318**	0.280	1.378	0.08	**2.196**	0.133	0.322	0.001
Virt6	− **1.970**	− 0.200	− 0.628	− 0.05	− 1.589	− 0.096	**2.124**	0.171	− 0.351	− 0.050
*Ensemble SPI*										
Accra	**1.988**	0.001	1.009	0.001	0.47	0.001	− **3.821**	− 0.001	− **2.535**	− 0.001
Pokuase	− 1.414	− 0.001	**9.000**	0.003	− **1.652**	− 0.001	− **2.496**	− 0.001	− **3.040**	− 0.001
Tema	0.925	0.000	0.683	0.001	**7.717**	0.002	**2.185**	0.001	− **5.291**	− 0.002
Virt1	− **3.614**	− 0.001	1.194	0.001	**6.321**	0.002	0.430	0.002	**3.546**	0.001
Virt2	− **3.266**	− 0.001	0.948	0.001	− 0.087	0.002	**2.856**	0.001	**3.033**	0.001
Virt3	− **3.758**	− 0.001	0.971	0.001	0.0173	0.001	**2.923**	0.001	**3.256**	0.001
Virt4	− **2.644**	− 0.001	**1.751**	0.001	0.070	0.001	**5.782**	0.001	**3.236**	0.001
Virt5	− **2.412**	− 0.001	**1.779**	0.001	0.074	0.001	**5.837**	0.001	**3.258**	0.001
Virt6	− **1.966**	− 0.001	**2.716**	0.001	1.625	0.001	0.697	0.002	− **1.809**	− 0.001
*Ensemble SPEI*										
Accra	**1.755**	0.001	**2.092**	0.001	**2.410**	0.001	− **0.699**	− 0.001	**7.723**	0.002
Pokuase	− **10.553**	− 0.005	− 0.199	0.001	**4.128**	0.001	10.226	0.004	**4.633**	0.002
Tema	− 0.713	− 0.000	**10.041**	0.003	**2.563**	0.001	**7.360**	0.003	**3.791**	0.001
Virt1	− **4.855**	− 0.001	0.109	0.002	**5.898**	0.002	− 0.133	0.001	**3.888**	0.002
Virt2	− **1.758**	− 0.001	− 0.42	0.002	− 0.456	0.002	**3.457**	0.002	**2.929**	0.001
Virt3	− **1.779**	− 0.001	− 1.002	0.001	− 0.0536	0.001	**3.173**	0.001	**3.201**	0.001
Virt4	− **3.986**	− 0.001	0.973	0.001	− 0.659	0.001	**6.559**	0.002	**3.998**	0.002
Virt5	− **3.709**	− 0.001	0.787	0.001	− 0.571	0.001	**2.419**	0.001	**4.911**	0.001
Virt6	− **3.087**	− 0.001	− **2.727**	− 0.001	− **3.500**	− 0.001	− **8.994**	− 0.003	− **1.663**	− 0.001
*Ensemble CWD*										
Accra	− 0.390	0.000	− **2.021**	0.000	− 1.353	0.000	0.410	0.001	**2.032**	0.001
Pokuase	0.830	0.000	− **2.047**	0.000	**1.681**	0.000	**1.864**	0.000	**2.419**	0.001
Tema	− **1.987**	0.000	− 1.513	0.000	**1.829**	0.000	1.077	0.000	**2.127**	0.002
Virt1	**1.971**	0.000	− 0.957	0.000	− **2.800**	0.000	− **2.800**	0.000	0.892	0.000
Virt2	1.002	0.000	− **4.752**	− 0.025	1.583	0.000	1.082	0.000	− 0.625	0.000
Virt3	1.009	0.000	− **2.997**	0.000	**1.724**	0.000	− 0.362	0.000	− 1.237	0.000
Virt4	0.363	0.000	− **1.839**	0.000	0.890	0.000	**2.668**	0.027	0.992	0.000
Virt5	0.342	0.000	− **2.129**	0.000	0.959	0.000	1.406	0.000	1.536	0.000
Virt6	0.486	0.000	0.287	0.000	− **2.170**	0.000	− 0.278	0.000	− 0.465	0.000
*Ensemble PRCPTOT*										
Accra	0.878	0.743	0.505	0.221	0.811	1.161	− **2.005**	− 1.154	**5.354**	11.905
Pokuase	− **1.836**	− 2.412	− **2.092**	− 1.158	**7.621**	7.188	− 0.914	− 1.059	**2.290**	3.113
Tema	− 0.211	− 0.498	1.375	0.82	0.1	0.873	**5.514**	4.373	**6.596**	4.073
Virt1	− **2.855**	− 1.841	**4.527**	4.169	1.043	0.695	1.483	1.448	**3.467**	2.507
Virt2	− **4.224**	− 1.732	**2.494**	1.2	0.257	0.097	0.695	0.561	**3.132**	1.438
Virt3	− **3.882**	− 1.947	**2.543**	1.393	0.498	0.117	0.76	0.593	**2.916**	1.691
Virt4	− **2.765**	− 0.937	**4.588**	2.597	1.6	1.087	1.101	1.013	**3.497**	2.059
Virt5	− 1.356	− 0.900	**4.447**	2.639	1.386	0.972	1.109	1.007	**3.475**	2.058
Virt6	− 1.195	− 1.193	**1.981**	1.252	− 0.283	− 0.115	**1.678**	1.69	− 0.184	− 0.069
*Ensemble R20mm*										
Accra	0.892	0.000	0.195	0.000	**3.416**	0.049	1.463	0.000	− 0.377	− 0.001
Pokuase	− 1.268	− 0.028	**4.488**	0.034	0.959	0.000	**7.359**	0.096	**4.047**	0.047
Tema	0.825	0.000	0.751	0.000	1.494	0.000	**2.052**	0.001	**3.573**	0.048
Virt1	0.066	0.000	**5.593**	0.056	**2.043**	0.043	1.993	0.000	0.629	0.000
Virt2	− **3.883**	− 0.080	**1.992**	0.000	**7.294**	0.036	0.912	0.000	0.762	0.000
Virt3	− **7.043**	− 0.120	**2.509**	0.000	**7.607**	0.042	1.057	0.000	− 0.028	0.000
Virt4	− **3.703**	− 0.083	**4.588**	0.031	**6.369**	0.059	− 0.169	0.000	1.090	0.000
Virt5	− **3.527**	− 0.077	**6.705**	0.048	**7.244**	0.060	1.433	0.012	− 0.067	0.000
Virt6	− **2.510**	− 0.032	0.779	0.000	1.114	0.000	1.110	0.000	0.237	0.000
*Ensemble R99pTOT*										
Accra	1.460	0.000	**2.342**	0.034	0.462	0.001	1.486	0.001	0.176	0.002
Pokuase	0.023	0.000	**2.118**	0.001	1.275	0.000	**1.746**	0.001	**1.987**	0.001
Tema	0.559	0.000	**2.104**	0.001	− 0.615	0.000	**2.156**	0.002	1.202	0.000
Virt1	− **1.777**	− 0.088	0.148	0.000	0.904	0.000	1.380	0.000	**2.041**	0.000
Virt2	− **2.594**	− 0.049	0.442	0.000	− 0.443	0.000	− 0.520	0.000	**2.037**	0.000
Virt3	− **3.618**	− 0.106	0.822	0.000	− 0.705	0.000	0.000	0.000	**2.093**	0.002
Virt4	− **5.240**	− 0.213	1.582	0.000	− 0.139	0.000	**1.871**	0.000	**2.200**	0.000
Virt5	− **3.093**	− 0.163	0.806	0.000	0.938	0.000	**1.975**	0.000	**2.114**	0.000
Virt6	− **1.830**	0.000	**2.390**	0.000	0.207	0.000	0.565	0.000	1.174	0.000
*Ensemble R99p*										
Accra	**1.647**	0.001	0.724	0.001	**1.826**	0.001	1.201	0.001	**2.065**	0.003
Pokuasea	− 1.038	0.000	1.230	0.000	**2.123**	0.001	− 1.339	0.000	**2.239**	0.001
Tema	0.599	0.000	− 0.239	0.000	**2.225**	0.002	0.478	0.000	**2.360**	0.002
Virt1	− 1.475	0.000	0.525	0.000	1.509	0.000	0.778	0.000	**1.855**	0.002
Virt2	− **2.798**	− 0.618	− 0.643	0.000	0.306	0.000	0.577	0.000	**2.607**	0.146
Virt3	− **4.519**	− 1.420	− 0.822	0.000	0.773	0.000	1.000	0.000	**2.579**	0.187
Virt4	− 1.547	0.000	0.294	0.000	**1.648**	0.000	1.299	0.000	**2.083**	0.000
Virt5	− 1.442	0.000	1.237	0.000	0.758	0.000	**1.707**	0.000	**1.995**	0.000
Virt6	− **2.497**	0.000	0.354	0.000	**2.393**	0.000	0.746	0.000	1.412	0.000
*Ensemble Rx5days*										
Accra	− **1.856**	− 0.496	0.379	0.146	**1.601**	0.066	**1.947**	0.337	**4.140**	0.700
Pokuse	0.324	0.037	0.482	0.103	− **2.792**	− 0.664	1.400	0.314	**5.344**	0.903
Tema	0.186	0.103	**2.006**	0.246	0.348	0.070	**6.877**	1.691	**6.381**	0.904
Virt1	− **2.073**	− 0.396	− 0.424	− 0.076	− 0.328	− 0.036	1.171	0.267	**3.031**	0.440
Virt2	− **3.804**	− 0.361	− 1.240	− 0.127	− 0.474	− 0.062	1.404	0.137	1.637	0.137
Virt3	**5.999**	− 0.480	− 1.506	− 0.122	− 0.504	− 0.063	**2.196**	0.219	1.549	0.144
Virt4	− **1.960**	− 0.205	− 0.108	− 0.038	0.000	− 0.001	**1.977**	0.232	**3.542**	0.380
Virt5	− 0.827	− 0.205	0.665	0.121	2.046	0.230	**2.046**	0.230	**3.640**	0.432
Virt6	− **2.059**	− 0.277	− **2.883**	− 0.360	1.464	0.322	0.500	0.093	**3.016**	0.394
*Ensemble Tx10p*										
Accra	− **7.462**	− 0.131	− **4.074**	− 0.465	− **4.420**	− 0.441	− **4.562**	− 0.450	− **5.427**	− 0.591
Pokuase	− **3.746**	− 0.349	− **5.567**	− 0.470	− **5.881**	− 0.505	− **6.925**	− 0.542	− **7.001**	− 0.594
Tema	− **3.579**	− 0.248	− **4.984**	− 0.409	− **5.748**	− 0.491	− **7.110**	− 0.536	− **6.711**	− 0.571
Virt1	− **5.505**	− 0.273	− **7.281**	− 0.566	− **7.318**	− 0.572	− **5.613**	− 0.392	− **7.104**	− 0.504
Virt2	− **3.118**	− 0.185	− **5.513**	− 0.389	− **7.186**	− 0.505	− **7.304**	− 0.553	− **7.311**	− 0.569
Virt3	− **2.670**	− 0.151	− **7.611**	− 0.571	− **7.328**	− 0.579	− **5.449**	− 0.443	− **7.397**	− 0.507
Virt4	− **5.694**	− 0.301	− **7.363**	− 0.564	− **7.208**	− 0.572	− **5.543**	− 0.387	− **7.313**	− 0.498
Virt5	**8.095**	0.288	− **6.769**	− 0.550	− **6.912**	− 0.487	− **7.745**	− 0.562	− **5.455**	− 0.334
Virt6	**8.499**	0.318	− **3.317**	− 0.407	− **4.221**	− 0.438	− **5.251**	− 0.542	− **4.246**	− 0.422
*Ensemble Tx90p*										
Accra	**9.141**	0.184	**4.027**	0.436	**5.199**	0.482	**3.981**	0.436	**10.035**	0.449
Poku	**3.990**	0.124	**4.730**	0.530	**5.994**	0.544	**5.015**	0.626	**21.330**	0.446
Tema	**6.183**	0.276	**4.742**	0.508	**5.974**	0.511	**4.921**	0.588	**20.061**	0.404
Virt1	**7.638**	0.278	**4.848**	0.556	**6.623**	0.526	**4.938**	0.563	**15.234**	0.376
Virt2	**4.184**	0.236	**4.747**	0.543	**6.486**	0.518	**4.902**	0.566	**16.066**	0.371
Virt3	**3.677**	0.373	**4.767**	0.536	**6.224**	0.504	**5.058**	0.577	**13.018**	0.422
Virt4	**7.861**	0.322	**4.842**	0.554	**6.551**	0.519	**5.073**	0.584	**14.246**	0.376
Virt5	**8.095**	0.288	**5.132**	0.607	**6.317**	0.500	**4.940**	0.564	**10.836**	0.348
Virt6	**8.499**	0.318	**3.676**	0.289	**4.917**	0.522	**3.253**	0.226	**6.069**	0.391
*Ensemble Tn10p*										
Accra	− **8.263**	− 0.209	− **3.210**	− 0.273	− **3.817**	− 0.301	− **3.895**	− 0.313	− **4.111**	− 0.326
Poku	− 1.098	− 0.035	− **5.171**	− 0.540	− **4.357**	− 0.434	− **3.899**	− 0.386	− **4.278**	− 0.485
Tema	− **24.979**	− 0.272	− **4.882**	− 0.444	− **5.145**	− 0.521	− **5.418**	− 0.573	− **4.914**	− 0.591
Virt1	− **2.938**	− 0.232	− **3.753**	− 0.411	− **4.366**	− 0.467	− **5.139**	− 0.534	− **4.577**	− 0.507
Virt2	− **3.378**	− 0.280	− **3.653**	− 0.384	− **4.385**	− 0.480	− **5.132**	− 0.514	− **4.552**	− 0.513
Virt3	− **3.687**	− 0.339	− **3.862**	− 0.366	− **4.243**	− 0.467	− **4.702**	− 0.502	− **5.179**	− 0.535
Virt4	− **3.309**	− 0.272	− **3.555**	− 0.374	− **4.194**	− 0.464	− **4.726**	− 0.518	− **4.080**	− 0.384
Virt5	− **3.595**	− 0.290	− **5.091**	− 0.516	− **4.320**	− 0.471	− **3.647**	− 0.377	− **4.702**	− 0.505
Virt6	− **3.803**	− 0.293	− **3.248**	− 0.288	− **4.222**	− 0.405	− **4.592**	− 0.435	− **3.906**	− 0.311
*Ensemble Tn90p*										
Accra	**6.158**	0.348	**8.585**	0.420	**4.837**	0.478	**3.511**	0.184	**3.585**	0.283
Poku	**5.221**	0.321	**28.263**	0.500	**4.951**	0.577	**4.633**	0.557	**3.753**	0.373
Tema	**6.183**	0.276	**21.699**	0.461	**4.982**	0.555	**3.947**	0.427	**4.470**	0.539
Virt1	**9.000**	0.346	**18.136**	0.433	**4.828**	0.582	**3.748**	0.420	**4.342**	0.483
Virt2	**5.740**	0.283	**16.805**	0.424	**4.775**	0.558	**4.334**	0.459	**3.770**	0.433
Virt3	**5.618**	0.285	**8.812**	0.440	**4.957**	0.578	**3.748**	0.418	**4.087**	0.398
Virt4	**9.137**	0.370	**20.564**	0.436	**5.314**	0.580	**3.921**	0.527	**4.156**	0.396
Virt5	**11.429**	0.396	**19.551**	0.433	**4.861**	0.563	**4.062**	0.386	**3.743**	0.444
Virt6	**10.917**	0.398	**6.330**	0.371	**4.559**	0.467	**3.413**	0.191	**3.309**	0.198
*Ensemble TXx*										
Accra	**5.167**	0.015	**5.635**	0.018	**19.331**	0.038	**13.663**	0.050	**16.540**	0.052
Poku	0.962	0.000	**6.086**	0.015	**16.343**	0.025	**12.538**	0.030	**16.885**	0.042
Tema	**2.430**	0.023	**6.602**	0.012	**17.752**	0.023	**14.121**	0.025	**15.656**	0.036
Virt1	**2.362**	0.022	**11.758**	0.022	**14.778**	0.037	**9.907**	0.033	**9.044**	0.060
Virt2	0.021	0.002	**17.509**	0.023	**15.976**	0.033	**11.133**	0.029	**16.543**	0.057
Virt3	0.431	0.010	**6.464**	0.027	**17.662**	0.036	**10.733**	0.031	**20.159**	0.060
Virt4	**2.566**	0.017	**14.988**	0.021	**14.44**	0.034	**11.297**	0.028	**15.272**	0.056
Virt5	1.083	0.007	**7.478**	0.014	**10.830**	0.037	**9.885**	0.041	**13.546**	0.050
Virt6	0.512	0.003	**6.268**	0.034	**20.971**	0.083	**19.741**	0.096	**24.379**	0.112
*Ensemble TNx*										
Accra	0.658	0.004	**8.562**	0.090	**13.542**	0.154	**15.798**	0.213	**18.415**	0.200
Poku	0.212	0.000	**7.042**	0.035	**18.339**	0.071	**16.103**	0.089	**15.316**	0.092
Tema	**3.947**	0.033	**10.746**	0.019	**14.822**	0.039	**13.063**	0.055	**18.341**	0.055
Virt1	**7.225**	0.029	**3.947**	0.019	**20.358**	0.042	**12.674**	0.055	**17.491**	0.058
Virt2	**6.043**	0.025	**4.114**	0.018	**18.182**	0.040	**12.057**	0.052	**20.887**	0.053
Virt3	**6.179**	0.027	**5.848**	0.021	**19.930**	0.040	**11.831**	0.053	**17.252**	0.058
Virt4	**8.431**	0.029	**3.780**	0.016	**11.446**	0.039	**12.334**	0.052	**18.072**	0.058
Virt5	**7.921**	0.030	**3.896**	0.017	**21.901**	0.041	**10.245**	0.050	**17.021**	0.058
Virt6	**6.935**	0.024	**7.794**	0.035	**14.120**	0.067	**12.547**	0.079	**12.021**	0.087
*Ensemble WSDI*										
Accra	**2.512**	0.000	**2.536**	0.000	**2.670**	0.001	**2.881**	0.001	**2.991**	0.002
Poku	0.811	0.000	**2.047**	0.000	**2.850**	0.000	**2.707**	0.001	**2.325**	0.001
Tema	1.346	0.000	**2.373**	0.000	**2.860**	0.000	**2.680**	0.001	**2.368**	0.001
Virt1	**1.688**	0.000	**2.551**	0.000	**2.743**	0.000	**2.424**	0.000	**2.286**	0.000
Virt2	0.674	0.000	**2.480**	0.000	**2.546**	0.000	**2.407**	0.000	**0.023**	0.000
Virt3	1.622	0.000	**2.404**	0.000	**2.540**	0.000	**2.538**	0.000	**2.294**	0.000
Virt4	1.476	0.000	**2.608**	0.000	**2.620**	0.000	**2.391**	0.000	**2.312**	0.000
Virt5	**1.677**	0.000	**2.389**	0.000	**2.441**	0.000	**2.513**	0.000	**2.936**	0.000
Virt6	1.615	0.000	**4.430**	0.271	**2.841**	0.000	**2.977**	0.000	**2.514**	0.000
*Ensemble CDDcold*										
Accra	**13.731**	7.234	**6.404**	10.788	**21.178**	17.166	**12.523**	21.843	**14.723**	24.515
Poku	**2.467**	1.913	**7.571**	6.480	**18.314**	16.191	**12.050**	20.308	**15.331**	22.237
Tema	**6.865**	10.392	**7.505**	7.831	**19.388**	10.580	**13.708**	13.241	**14.070**	14.042
Virt1	**6.976**	8.355	**7.505**	7.831	**21.222**	13.208	**10.414**	16.395	**11.650**	18.606
Virt2	**4.405**	7.117	**7.586**	7.284	**22.721**	12.120	**10.291**	15.090	**11.508**	17.190
Virt3	**4.447**	6.851	**10.230**	9.139	**22.327**	12.704	**10.353**	15.828	**10.753**	18.181
Virt4	**7.739**	8.737	**7.568**	7.181	**17.224**	11.556	**12.950**	14.693	**10.846**	17.253
Virt5	**7.579**	8.566	**7.111**	7.405	**25.027**	12.857	**10.914**	16.484	**10.463**	18.618
Virt6	**8.087**	8.782	**6.235**	12.593	**21.082**	19.748	**11.557**	24.745	**11.291**	27.576

NB: The values in bold represent significance at 5% level and 95% confidence level from the MMTK test and Sen’s slope estimator. However, values not in bold are insignificant at the same significance and confidence level

## Appendix 2

See Fig. 14.
